# Single Atom Iron‐Doped Graphic‐Phase C_3_N_4_ Semiconductor Nanosheets for Augmented Sonodynamic Melanoma Therapy Synergy with Endowed Chemodynamic Effect

**DOI:** 10.1002/advs.202302579

**Published:** 2023-06-06

**Authors:** Guiying Feng, Hui Huang, Min Zhang, Zhuole Wu, Dandan Sun, Qiqing Chen, Dayan Yang, Yuanyi Zheng, Yu Chen, Xiangxiang Jing

**Affiliations:** ^1^ Department of Ultrasonography Hainan General Hospital/Hainan Affiliated Hospital of Hainan Medical University 570311 Haikou P. R. China; ^2^ Materdicine Lab, School of Life Sciences Shanghai University Shanghai 200444 P. R. China; ^3^ Department of Ultrasound in Medicine Shanghai Jiao Tong University Affiliated Sixth People’s Hospital, Shanghai Institute of Ultrasound in Medicine 200032 Shanghai P. R. China

**Keywords:** carbon nitride, chemoreactive medicine, reactive oxygen species, semiconductor, single atom

## Abstract

Sonodynamic therapy (SDT) is a non‐invasive therapeutic modality with high tissue‐penetration depth to induce reactive oxygen species (ROS) generation for tumor treatment. However, the clinical translation of SDT is restricted seriously by the lack of high‐performance sonosensitizers. Herein, the distinct single atom iron (Fe)‐doped graphitic‐phase carbon nitride (C_3_N_4_) semiconductor nanosheets (Fe‐C_3_N_4_ NSs) are designed and engineered as chemoreactive sonosensitizers to effectively separate the electrons (e^−^) and holes (h^+^) pairs, achieving high yields of ROS generation against melanoma upon ultrasound (US) activation. Especially, the single atom Fe doping not only substantially elevates the separation efficiency of the e^−^‐h^+^ pairs involved in SDT, but also can serve as high‐performance peroxidase mimetic enzyme to catalyze the Fenton reaction for generating abundant hydroxyl radicals, therefore synergistically augmenting the curative effect mediated by SDT. As verified by density functional theory simulation, the doping of Fe atom significantly promotes the charge redistribution in the C_3_N_4_‐based NSs, which improves their synergistic SDT/chemodynamic activities. Both the in vitro and in vivo assays demonstrate that Fe‐C_3_N_4_ NSs feature an outstanding antitumor effect by aggrandizing the sono‐chemodynamic effect. This work illustrates a unique single‐atom doping strategy for ameliorating the sonosensitizers, and also effectively expands the innovative anticancer‐therapeutic applications of semiconductor‐based inorganic sonosensitizers.

## Graphical abstract

The distinct single atom iron (Fe)‐doped graphitic‐phase carbon nitride (C_3_N_4_) semiconductor nanosheets (Fe‐C_3_N_4_ NSs) can achieve high yields of reactive oxygen species generation upon ultrasound activation for synergistic sonodynamic therapy and chemodynamic therapy for melanoma therapy.

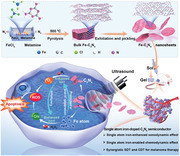

## 1 Introduction

Malignant cancer is the second serious cause of death in humans,^[^ ^1^ ^]^ and the current therapeutic strategies mainly include surgical operation, radiotherapy, and chemotherapy, which generally have limitations of incomplete removal of lesions and inevitable side effects.^[^ ^2^ ^]^ In order to overcome the limitations of traditional treatments, the nanodynamic therapy (NDT) that is created or assisted by exogenous stimuli or endogenous triggers combined with nanomaterials, has been recently developed as diverse dynamic therapy modalities for efficient tumor treatment, predominantly including chemodynamic therapy (CDT),^[^ ^3^ ^]^ sonodynamic therapy (SDT),^[^ ^4^ ^]^ electrodynamic therapy,^[^ ^5^ ^]^ thermodynamic therapy,^[^ ^6^ ^]^ piezoelectric dynamic therapy,^[^ ^7^ ^]^ pyroelectric dynamic therapy,^[^ ^8^ ^]^ and radiodynamic therapy.^[^ ^9^ ^]^

Ultrasound (US) is an external physical activator with noninvasiveness and high tissue‐penetrating property, which has been extensively used in clinic for diagnosis and treatment of diseases. SDT, as one kind of NDT, refers to producing reactive oxygen species (ROS)‐related cytotoxicity to combat various diseases by using ultrasonic waves to activate sonosensitizers.^[^ ^4^, ^7^, ^10^ ^]^ The traditional sonosensitizers mainly include organic sonosensitizers (e.g., hematoporphyrin, photofrin, hematoporphyrin mono‐methyl ether (HMME), photoporphyrin IX, ATX‐70, etc.)^[^ ^11^ ^]^ and inorganic sonosensitizers (e.g., titanium dioxide (TiO_2_)^[^ ^12^ ^]^ and TiO_2_‐based hybrid nanoparticles (NPs),^[^ ^13^ ^]^ oxygen‐deficient NPs,^[^ ^14^ ^]^ fullerenes,^[^ ^15^ ^]^ silver selenide (Ag_2_Se) QDs,^[^ ^16^ ^]^ and black phosphorus (BP) NSs,^[^ ^17^ ^]^ etc.). These sonosensitizers usually suffer from several disadvantages, such as high phototoxicity, low sonodynamic efficiency, and potential long‐term retention within the body.^[^ ^4^, ^18^ ^]^ Therefore, it is highly desirable to develop high‐performance sonosensitizers with favorable biocompatibility for disease treatment. Graphitic‐phase carbon nitride (C_3_N_4_) are semiconductor photocatalysts consisting of C and N elements, which features high separation efficiency of electron (e^−^)‐hole (h^+^) and production of ROS upon UV–visible light irradiation.^[^ ^19^ ^]^ Owing to their distinct features, such as facile synthesis, high stability, narrow and controllable band gap (2.7 eV), and low toxicity,^[^ ^19^, ^20^ ^]^ C_3_N_4_ nanosheets (NSs) exhibit considerable potential in the field of SDT for tumor therapy. However, the practical applications of C_3_N_4_ catalysts are still restricted due to their undesirable drawbacks including unsatisfactory water‐solubility, relatively large particle size, low electrical conductivity, and rapid recombination of e^−^and h^+^ pairs.^[^ ^20^, ^21^ ^]^ Metal element doping (e.g., ferrum (Fe),^[^ ^22^ ^]^ copper (Cu),^[^ ^23^ ^]^ manganese (Mn),^[^ ^24^ ^]^ etc.) can narrow the band gap of C_3_N_4_ and promote the separation of e^−^ and h^+^ pairs to enhance the generation efficiency of ROS owing to the *π*‐electron delocalization of the electronic band structure of C_3_N_4_,^[^ ^22^, ^25^ ^]^ which is a facile and effective modification method to improve the sonocatalytic activity of C_3_N_4_ catalysts. Simultaneously, C_3_N_4_ catalysts also act as the desirable support for metal ion complexation or doping by taking advantage of N‐coordinated holes and high‐density uniform “sixfold cavities.”^[^ ^25a^ ^]^

CDT based on Fenton or Fenton‐like reaction is defined as the process of generating ROS by converting hydrogen peroxide (H_2_O_2_) into hydroxyl radical (∙OH) in the mildly acidic tumor microenvironment (TME) with the assistance of Fenton or Fenton‐like catalysts.^[^ ^4^, ^26^ ^]^ At present, a variety of iron‐based Fenton catalysts have been successfully developed, including zero‐valent iron,^[^ ^27^ ^]^ iron minerals and iron (hydr)oxide,^[^ ^28^ ^]^ multimetallic and supported iron‐based materials,^[^ ^29^ ^]^ etc. However, these iron‐involving catalysts have some shortcomings of large size, diffusion resistance, and especially the low active site dispersion to fail in achieving satisfying ROS production.^[^ ^26b^ ^]^ Therefore, it is highly important to explore alternative Fenton nanocatalysts with ever‐higher active‐site and catalytic activities. In general, biomedical applications and potential toxicity usually depend on the metal atomic sites in nanocatalysts, thus how to maximize the utilization efficiency of metal atoms is the primary issue.^[^ ^30^ ^]^ Due to the superiority of higher atom utilization, unique low‐coordination environments, and electronic structure,^[^ ^25^, ^26^, ^31^ ^]^ single‐atom iron catalysts have exhibited excellent catalytic performances and achieved satisfying tumor‐therapeutic effects at relatively low concentrations. More importantly, carbon‐based materials are suitable and promising supports to anchor the isolated Fe single atoms because of Fe‐N coordination, which elevates the utilization of the iron active site.^[^ ^22^, ^31^ ^]^

**Scheme 1:**
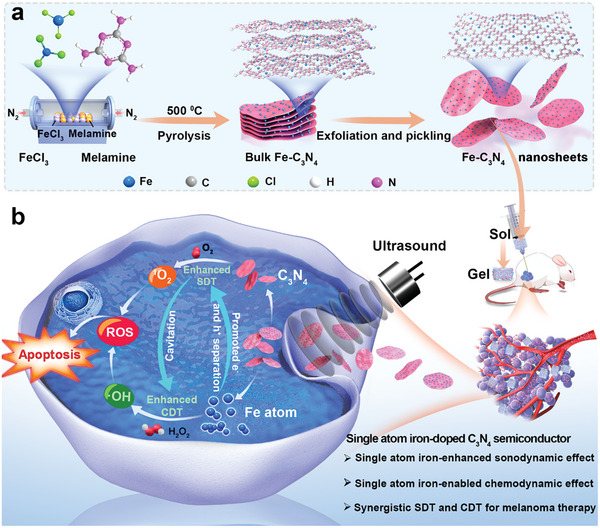
The design principle. a) The construction of Fe‐C_3_N_4_ NSs. b) Schematic illustration of the underlying therapeutic mechanisms of US‐activated Fe‐C_3_N_4_ for synergistic and bilateral enhancement of SDT and CDT under TME.

In this work, we elaborated and engineered the distinct chemoreactive nanosonosensitizers (Fe‐C_3_N_4_ NSs) via doping single atom iron into the C_3_N_4_ nanocarrier. Compared with pure C_3_N_4_ NSs, the Fe‐C_3_N_4_ nanosystem exhibits higher separation efficiency of e^−^‐h^+^ pairs to generate abundant ROS for melanoma treatment under US activation. Furthermore, Fe‐C_3_N_4_ NSs also possess high‐performance peroxidase (POD)‐like activity to conduct Fenton catalytic reaction to transform H_2_O_2_ into cytotoxic ∙OH in the TME. In the meantime, Fe‐C_3_N_4_ NSs under US activation can realize the synergistic enhancement of SDT and CDT to generate sufficient ROS for cancer therapy (**Scheme** 1). The mechanism of synergistically enhanced SDT and Fenton catalytic reaction of the Fe‐C_3_N_4_ NSs triggered by US is attributed to the significant charge redistribution after doping single Fe atoms to the C_3_N_4_ matrix, as verified by the density functional theory (DFT). The apparent melanoma suppression by the sono‐chemodynamic effect of Fe‐C_3_N_4_ NSs is confirmed by both in vitro and in vivo experiments, probably involving the biological mechanisms of regulating the oxidative stress response, ROS generation, and apoptosis according to the RNA sequencing (RNAseq) results. This strategy based on the single atom iron‐doped C_3_N_4_ nanosystem provides a paradigm for semiconductor‐based nanosonosensitizers to augment the ROS production for oncotherapy.

## 2 Results and Discussion

### 2.1 Synthesis and Characterization of Fe‐C_3_N_4_ NSs

The design and fabrication of Fe‐C_3_N_4_ NSs are shown in Scheme 1a. In detail, the pre‐fabricated Fe‐doped C_3_N_4_ (bulk Fe‐C_3_N_4_) were initially synthesized via a modified one‐step pyrolysis method,^[^ ^25a^ ^]^ in which melamine (MA) and anhydrous ferric chloride (FeCl_3_) were calcined under 500 °C. Subsequently, single‐ or few‐layer Fe‐C_3_N_4_ NSs were constructed by ultrasonic exfoliation, and the undoped‐Fe substances during the synthesis process were removed by pickling method.^[^ ^20c^ ^]^

**Figure 1:**
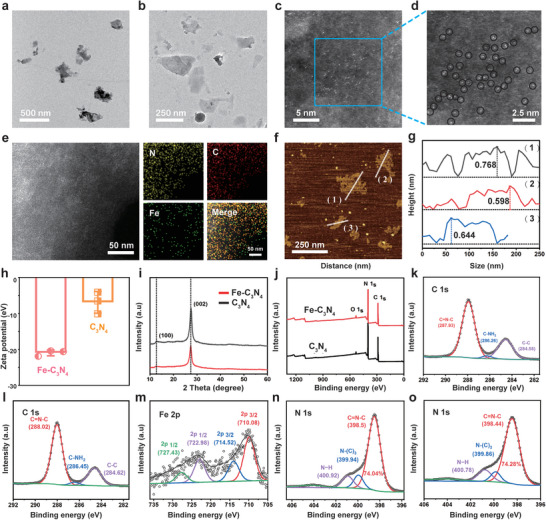
Characterization of Fe‐C_3_N_4_ NSs. TEM image of a) C_3_N_4_ and b) Fe‐C_3_N_4_ NSs. c,d) HAADF‐STEM of the Fe‐C_3_N_4_ NSs and the single Fe atoms labeled with black circles. e) HAADF‐STEM image of Fe‐C_3_N_4_ NSs and the corresponding elemental mapping of, N, C, and Fe. f) AFM image and g) the corresponding height of Fe‐C_3_N_4_ NSs. h) Zeta potential of Fe‐C_3_N_4_ and C_3_N_4_ NSs. i) XRD patterns of C_3_N_4_ and Fe‐C_3_N_4_ NSs. j) XPS survey spectrum of Fe‐C_3_N_4_ and C_3_N_4_ NSs. C 1s XPS spectrum of k) Fe‐C_3_N_4_ and l) C_3_N_4_ NSs. m) Fe 2p XPS spectrum of Fe‐C_3_N_4_ NSs. N 1s XPS spectrum of n) C_3_N_4_ and o) Fe‐C_3_N_4_ NSs.

The scanning electron microscopy (SEM) images showed that the bulk Fe‐C_3_N_4_ was comprised of numerous compact and variably‐sized pieces, which was similar to the structure of the pre‐fabricated C_3_N_4_ (bulk C_3_N_4_), and the elemental mapping clearly displayed the coexistence of Fe, carbon (C) and nitrogen (N) elements in bulk Fe‐C_3_N_4_ (Figures S1 and S2, Supporting Information). These bulk products could be further developed into Fe‐C_3_N_4_ and C_3_N_4_ NSs after ultrasonic exfoliation, respectively, as observed by transmission electron microscopy (TEM) (**Figure** **1**a,b). The atomic microstructure of Fe‐C_3_N_4_ NSs was characterized by high‐angle annular dark field‐scanning TEM (HAADF‐STEM). According to the contrast difference between metal atoms with N/C atoms,^[^ ^22^, ^25^ ^]^ it is found that a number of ultra‐small Fe atoms labeled with the black circles were uniformly dispersed in the C_3_N_4_ matrix (Figure 1c,d). The energy dispersive X‐ray spectroscopy element mapping of the Fe‐C_3_N_4_ NSs further testified that a few scattered Fe atoms were distributed between C and N elements (Figure 1e; Figure S3, Supporting Information), and the Fe loading efficiency in the Fe‐C_3_N_4_ NSs was estimated to be 0.16 wt% through inductively coupled plasma‐atomic emission spectrometer. Atomic force microscope (AFM) revealed that the height of Fe‐C_3_N_4_ NSs was about 0.670 nm, which was less than that of C_3_N_4_ NSs (0.908 nm) (Figure 1f,g; Figure S4, Supporting Information). Given that both the theoretical thickness and interlayer spacing of the C_3_N_4_ monolayer were ≈0.3 nm,^[^ ^19^, ^32^ ^]^ the as‐synthesized Fe‐C_3_N_4_ NSs probably possessed single‐ or few‐layer structures. After the incorporation of Fe atoms in the C_3_N_4_ NSs, the average hydrodynamic particle size was decreased from 301.1 to 253.2 nm (Figure S5, Supporting Information), and the zeta potential was changed from −6.9 to −21.0 mV (Figure 1h), which further confirmed the successful doping of single atom Fe in the sheet structure of C_3_N_4_. In addition, the Fe‐C_3_N_4_ NSs exhibited satisfactory stability in water and Roswell Park Memorial Institute 1640 Medium (RPMI 1640) for 24 h (Figure S6, Supporting Information).

X‐ray powder diffraction (XRD) was used to characterize the crystal structure of C_3_N_4_ and Fe‐C_3_N_4_ NSs (Figure 1i). Two apparent diffraction peaks, as observed at 13° and 27° in C_3_N_4_ NSs, were ascribed to the (100) and (002) planes, which equaled to the planar configuration of tris‐triazine units and interlayer accumulation of aromatic units, respectively (JCPDS87‐1526).^[^ ^33^ ^]^ Compared with C_3_N_4_, the crystallinity of (100) and (002) planes in Fe‐C_3_N_4_ NSs became weaker, indicating that the presence of single‐atom Fe affected the pristine structure of the C_3_N_4_. Additionally, there was no signal associated with Fe‐derived metal nanoparticles.^[^ ^22^ ^]^ As presented in Figure 1j, the survey scan spectra of the Fe‐C_3_N_4_ NSs directly visualized the coexistence of Fe, C, N, and O components without any other impurities. In particular, the C 1s X‐ray photoelectron spectroscopy (XPS) spectrum of Fe‐C_3_N_4_ catalysts showed that the binding energy of the triazine aromatic ring, C‐NH_2_ groups on the edges of heptazine units, and C‐C groups were all lower than those of C_3_N_4_ (Figure 1k,l), indicating the chemical environment of C species in Fe‐C_3_N_4_ NSs had been changed.^[^ ^25a^ ^]^ As displayed in the Fe 2p XPS spectrum of Fe‐C_3_N_4_ NSs, the dominant peaks at 710.08 and 722.98 eV corresponded to Fe 2p_3/2_ and Fe 2p_1/2_ of Fe (II), while the lower binding‐energy at 714.52 and 727.43 eV belonged to Fe 2p_3/2_ and Fe 2p_1/2_ of Fe (III) (Figure 1m).^[^ ^25a^ ^]^ The three typical peaks in the N 1s XPS spectrum of C_3_N_4_ were 398.5, 399.94, and 400.92 eV, corresponding to the sp^2^ C=N—C bonds in the tri‐s‐triazine (pyridinic‐N), sp^3^ tertiary nitrogen N−(C)_3_ groups and N−H groups, respectively (Figure 1n). Among them, the dominant C=N—C bonds of the N element could provide more sites for anchoring Fe atoms, because the N element with higher electrophilicity in the tri‐s‐triazine heteroring was more favorable to donate pairs of e^−^ to Fe atom.^[^ ^22^ ^]^ Therefore, the content of pyridinic‐N groups in Fe‐C_3_N_4_ was higher than that of C_3_N_4_ catalysts (74.28% versus 74.04%, respectively) (Figure 1n,o). Furthermore, the N 1s signals of Fe‐C_3_N_4_ were shifted to the lower binding energy compared with C_3_N_4_, proving the existence of Fe—N*_x_* bonding in the Fe‐C_3_N_4_ NSs.^[^ ^25a^ ^]^

### 2.2 In Vitro SDT and Fenton Activities of Fe‐C_3_N_4_ NSs under US Irradiation

**Figure 2:**
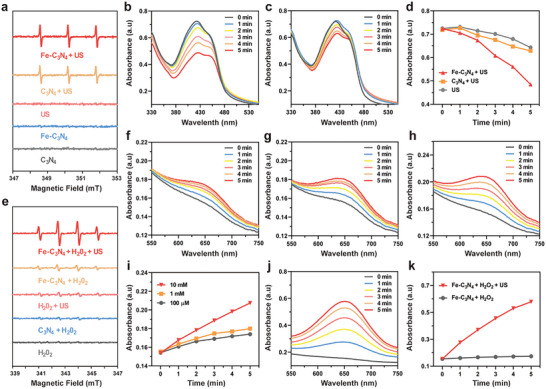
ROS generation of Fe‐C_3_N_4_ NSs. a) ^1^O_2_ generation by ESR spectroscopy using TEMP as the spin trap. The absorption intensities of DPBF in b) Fe‐C_3_N_4_ + US group and c) C_3_N_4_ + US group after different US durations (0, 1, 2, 3, 4, and 5 min). d) The absorption intensities of DPBF treated with Fe‐C_3_N_4_, C_3_N_4_, and DI‐water followed by US irradiation (0, 1, 2, 3, 4, and 5 min). e) ∙OH production by ESR spectroscopy using DMPO as the spin trap. The absorbance of TMB in the Fe‐C_3_N_4_ + H_2_O_2_ group with varying concentrations of H_2_O_2_, including f) 100 µm, g) 1 mm, and h) 10 mm buffered at pH = 5.4 within 5 min. i) Comparison of TMB oxidation treated with Fe‐C_3_N_4_ NSs in the elevated concentrations of H_2_O_2_ solution at pH = 5.4 after different durations (0, 1, 2, 3, 4, and 5 min). j) The absorption intensities of TMB in the Fe‐C_3_N_4_ + H_2_O_2_ + US group after different durations (0, 1, 2, 3, 4, and 5 min). k) Comparison of TMB absorption intensities of Fe‐C_3_N_4_ + H_2_O_2_ + US and Fe‐C_3_N_4_ + H_2_O_2_ at pH = 5.4 under US irradiation for different durations (0, 1, 2, 3, 4, and 5 min).

First, the SDT efficiency of Fe‐C_3_N_4_ NSs was evaluated via the typical electron spin resonance (ESR), in which 2, 2, 6, 6 tetramethyl‐4‐piperidone hydrochloride (TEMP) served as a spin trap to measure the singlet oxygen (^1^O_2_) generation. As shown in the ESR spectrum, the characteristic 1:1:1 triple splitting signal was notably observed in the Fe‐C_3_N_4_ + US and C_3_N_4_ + US groups (**Figure** **2**a), indicating that the C_3_N_4_‐based NSs could act as favorable nanosonosensitizers. To further evaluate the SDT performance of Fe‐C_3_N_4_ NSs, 1, 3‐diphenylisobenzofuran (DPBF) was used as the chemical probe to detect US‐triggered ^1^O_2_ production. The typical absorption peak (423 nm) of the DPBF gradually decreased by prolonging US irradiation duration, indicating the continuous ^1^O_2_ generation in the Fe‐C_3_N_4_ + US group (Figure 2b). In addition, compared with the US and C_3_N_4_ + US groups at the same concentration, the Fe‐C_3_N_4_ NSs exhibited a significantly higher oxidation rate of DPBF upon US irradiation (Figure 2c,d; Figure S7, Supporting Information), demonstrating that the SDT performance was substantially improved after the incorporation of single‐atom Fe into C_3_N_4_ NSs.

In order to identify the Fenton catalytic activity of Fe‐C_3_N_4_ NSs, 5,5‐dimethyl‐1‐ pyridine N‐oxide (DMPO) was used as a spin trap to assess the production of toxic hydroxyl radical (∙OH) using ESR. The ESR spectrum of Fe‐C_3_N_4_ + H_2_O_2_ and Fe‐C_3_N_4_ + H_2_O_2_ + US groups exhibited standard equidistant quartets (1:2:2:1) (Figure 2e), illustrating that Fe‐C_3_N_4_ NSs could act as the Fenton catalysts to transform H_2_O_2_ into ∙OH. Subsequently, we used 3, 3′, 5, 5′‐tetramethylbenzidine dihydrochloride (TMB) to further verify the ∙OH generation of Fe‐C_3_N_4_ NSs upon US irradiation. As shown in Figure 2f–i, the typical absorption peak of TMB (≈ 650 nm) representing ∙OH production elevated gradually with the prolongation of time and the increase of H_2_O_2_ concentration in the mildly acidic environment (pH 5.4), verifying that the single‐atom Fe in Fe‐C_3_N_4_ NSs possessed benign POD‐like catalytic activity to exert the Fenton reaction to catalyze H_2_O_2_ into ∙OH.^[^ ^34^ ^]^ In particular, all the absorption peaks of TMB in Fe‐C_3_N_4_ + H_2_O_2_ + US group after different US durations were higher than that of Fe‐C_3_N_4_ + H_2_O_2_ group (Figure 2j,k), proving that the Fenton performance of Fe‐C_3_N_4_ NSs could be dramatically enhanced by US irradiation.

### 2.3 Mechanism of SDT and Fenton Effect of Fe‐C_3_N_4_ NSs

**Figure 3:**
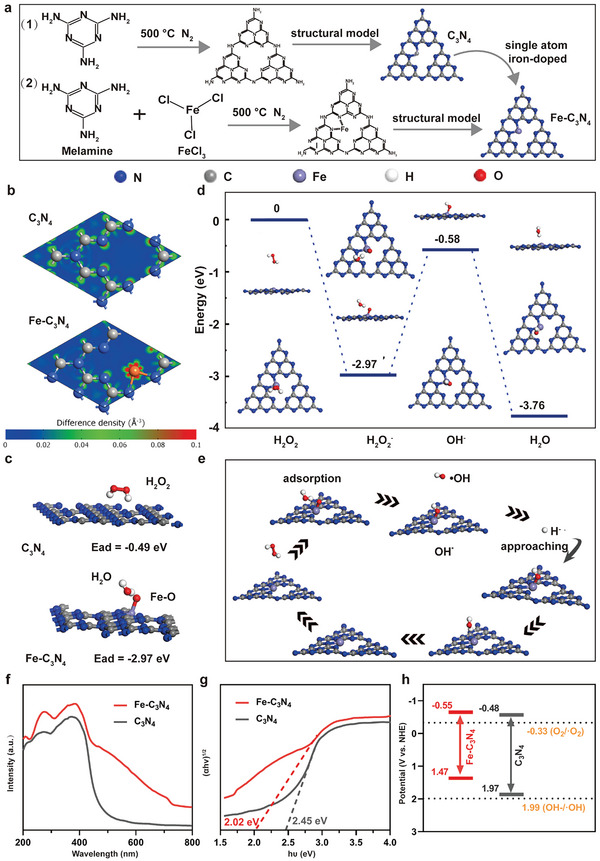
a) Schematic diagram of the synthetic process of C_3_N_4_ and Fe‐C_3_N_4_ NSs. b) Charge density differences of C_3_N_4_ and Fe‐C_3_N_4_ NSs. The charge depletion and accumulation regions are rendered in blue and red. c) Adsorption structures and adsorption energies of H_2_O_2_ adsorbed on C_3_N_4_ and Fe‐C_3_N_4_. d,e) Energy profile of POD‐like activity for Fe‐C_3_N_4_. f) UV–vis diffuses reflectance spectra, g) band gaps, and h) the energy band of C_3_N_4_ and Fe‐C_3_N_4_ NSs.

In order to reveal the potential mechanism of SDT and Fenton activities induced by Fe‐C_3_N_4_ NSs, we investigated the specifics of the Fe—N*_x_* bonding using the DFT and the remarkable variation of the band gap of C_3_N_4_‐based NSs. It is clear that the Fe‐C_3_N_4_ NSs were constructed by a synthetic process of the one‐step pyrolysis method. The C=N—C bonds in the triazine skeleton structure of C_3_N_4_ had successfully changed into Fe—N*_x_* bonding after the doping of Fe atoms into the C_3_N_4_ nanostructure (**Figure** **3**a). To shed light on the Fe—N*_x_* bonding behavior, the charge density difference based on the DFT of C_3_N_4_ and Fe‐C_3_N_4_ NSs was visualized in Figure 3b. It is found that the charge was accumulated on the Fe atom in the unit cell of Fe‐C_3_N_4_, and the charge density between C and N atoms became significantly lower than that in pure C_3_N_4_, indicating that the bond interaction between C and N attenuated while that between N and Fe strengthened. Therefore, the doping of the Fe atom in C_3_N_4_ promoted significant charge redistribution, making Fe‐C_3_N_4_ capture the US‐generated carriers and inhibit the recombination of e^−^‐h^+^ pairs effectively, which improved the separation efficiency of e^−^‐h^+^ and enhanced the SDT‐catalytic ability of the system. In addition, the charge accumulation located at the Fe atom, making it to be a Fenton‐catalytic active center, which potentially facilitated the H_2_O_2_ capture. The calculated adsorption energies of H_2_O_2_ on C_3_N_4_ and Fe‐C_3_N_4_ were −0.49 and −2.97 eV respectively, which confirmed the enhanced capture capacity on H_2_O_2_ of Fe‐C_3_N_4_ (Figure 3c). To further investigate the Fenton reaction mechanism, the energy profile of the POD‐like activity for Fe‐C_3_N_4_ NSs was calculated and illustrated in Figure 3d,e. First, the H_2_O_2_ molecule was facilely adsorbed at the Fe‐N*_x_* active site with an appropriate adsorption energy of −2.97 eV, and then one stable Fe—O bond accompanied by one adsorbed H_2_O was formed on the surface of Fe‐C_3_N_4_. Subsequently, it dissociated into a reactive ∙OH, as well as a hydroxyl adsorbed (OH*) at the Fe‐N*_x_* active site. Then, the OH* reacted with hydrogen ions (represented by protonated hydrogen atoms) to form H_2_O in the slightly acidic TME. The energy during the whole reaction process decreased by 3.76 eV, indicating the theoretical feasibility of the POD‐like activity after doping of the Fe atom to C_3_N_4_. Among them, the step of the reactive ∙OH generation was the determinant step, accompanied by an energy barrier of 2.39 eV. Finally, the Fe‐C_3_N_4_ recovered to the original state to participate in the next cycle of POD‐like reaction after desorption of H_2_O. In a word, the DFT results revealed that the enhanced POD‐like activity in Fe‐C_3_N_4_ derived from the charge redistribution as a consequence of the doping of the Fe atom.

Moreover, the solid ultraviolet spectra and Mott–Schottky plots were conducted to analyze the band alignment for clarifying the underlying SDT mechanism of Fe‐C_3_N_4_ NSs. As shown in Figure 3f,g, the band gap of Fe‐C_3_N_4_ NSs was 2.02 eV, which was narrower than 2.45 eV of pure C_3_N_4_, as calculated by the Tauc plot of the modified Kubelka–Munk function.^[^ ^4^, ^35^ ^]^ Furthermore, the flat band potential of Fe‐C_3_N_4_ and C_3_N_4_ NSs were −0.55 and −0.48 V versus the normal hydrogen electrode (NHE), respectively (Figure 3g,h; Figure S8, Supporting Information). Since the conduction bands (CB) of both catalysts were less than −0.33 V, e^−^ could transfer from the valence band (VB) to CB to reduce O_2_ to ∙O_2_^−^, and then ∙O_2_^−^ would be oxidized to ^1^O_2_ immediately due to the unstable nature of ∙O_2_^−^. Additionally, the CB energy of the Fe‐C_3_N_4_ system was higher than that of pure C_3_N_4_, therefore possessing stronger reducibility and more efficient separation of US‐generated carriers. Consequently, this interpretation regarding the mechanism of Fe‐C_3_N_4_‐based SDT was well consistent with that of the DFT computation in Figure 3b. However, the VB potential of Fe‐C_3_N_4_ and C_3_N_4_ NSs was lower than the redox potential of ∙OH/OH^−^ (1.99 V) (Figure 3h), which would fail to oxidize OH^−^ to ∙OH directly. Therefore, considering the analysis of DFT (Figure 3c–e), we speculated that the Fenton reaction mechanism of Fe‐C_3_N_4_ NSs resulted from the charge redistribution, which would potentially facilitate the transformation of H_2_O_2_ into ∙OH, and the reactivity could be further enhanced upon US irradiation.

In addition, the photoluminescence (PL) spectroscopy, time‐resolved photoluminescence spectroscopy, electrochemical impedance spectroscopy (EIS), and linear sweep voltammetry (LSV) were performed to analyze the enhanced catalytic mechanism. Compared with C_3_N_4_ NSs, the PL spectrum of Fe‐C_3_N_4_ NSs had a lower intensity, revealing the more ponderous recombination of charge carriers (Figure S9a, Supporting Information). Furthermore, a triexponential function was applied to analyze the lifetime of the carriers. The average lifetimes of the Fe‐C_3_N_4_ NSs (6.96 ns) were longer than that of C_3_N_4_ (2.41 ns), demonstrating that the recombination of e^−^ – h^+^ pairs in Fe‐C_3_N_4_ NSs was effectively constrained to initiate redox reactions (Figure S9b, Supporting Information). EIS was used to appraise the separation and migration capability of the e^−^ ‐ h^+^ pairs. As shown in Figure S10, Supporting Information, Fe‐C_3_N_4_ NSs exhibited a smaller semicircle radius of the impedance curve than C_3_N_4_, suggesting a lower resistance for charge transfer. The onset potential (*E*_onset_) and half‐wave potential (*E*_1/2_) in LSV curves reflected the oxygen reduction reaction (ORR) activity of the catalyst, and the higher *E*_onset_ and *E*_1/2_ value, the better catalyst performance was.^[^ ^36^ ^]^ From Figure S11a, Supporting Information, the onset potential (0.6663 V versus RHE) and half‐wave potential (0.5673 V versus RHE) of Fe‐C_3_N_4_ NSs were more positive than those of C_3_N_4_ (0.6583 V versus RHE and 0.5593 V versus RHE), suggesting that the active sites of iron served as a significant part to promote the process of catalytic activity. Furthermore, the Tafel slope was considered to be another crucial parameter for investigating ORR catalytic activity, which mainly investigated the reaction rate of the reaction.^[^ ^37^ ^]^ It was shown in Figure S11b, Supporting Information that the Tafel slope of the Fe‐C_3_N_4_ catalyst was ≈76.96 mV dec^−1^, lower than C_3_N_4_ NSs (137.29 mV dec^−1^). To sum up the above results, the doped Fe atoms in the C_3_N_4_ framework could elevate the separation efficiency of the e^−^‐h^+^ pairs and enhanced catalytic activity.

### 2.4 Bilaterally Enhanced SDT and CDT Effect of Fe‐C_3_N_4_ NSs In Vitro

**Figure 4:**
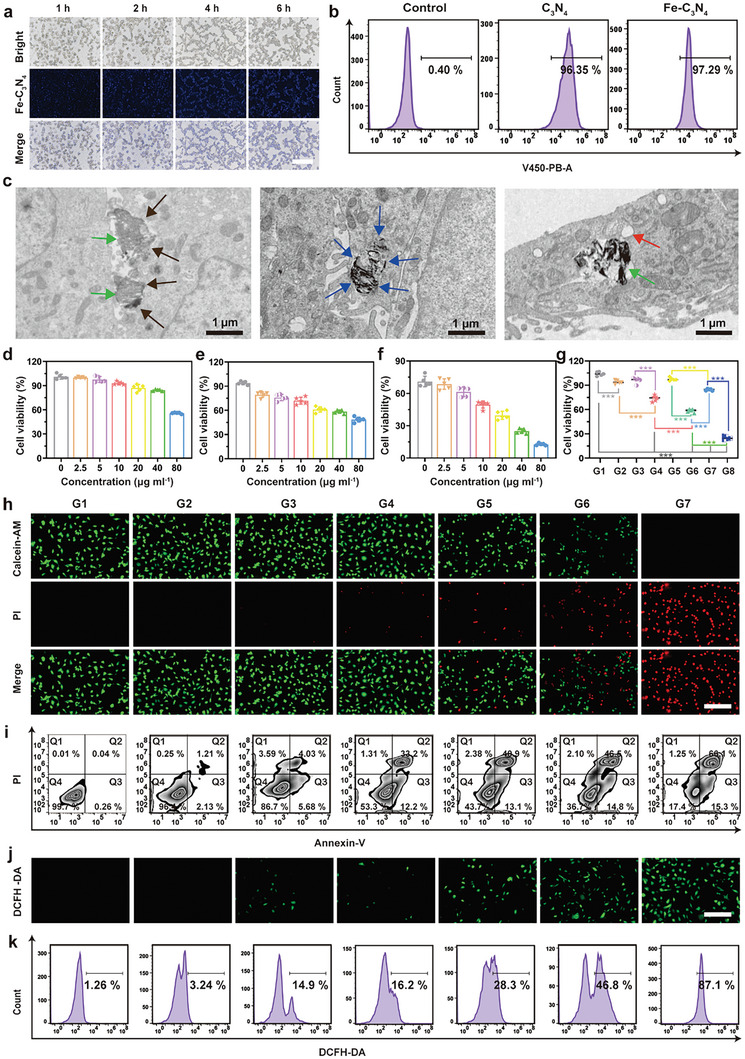
Bilateral enhancement of SDT/CDT for cancer therapy in vitro. a) Cellular uptake of Fe‐C_3_N_4_ NSs at 1, 2, 4, and 6 h observed by fluorescent microscope. Scale bar: 200 µm. b) Cellular uptake of C_3_N_4_ and Fe‐C_3_N_4_ NSs at 6 h analyzed by flow cytometry. c) Biological electron microscope images of B16F10 cells post co‐incubation with Fe‐C_3_N_4_ NSs. Fe‐C_3_N_4_ NSs labeled with the green arrow, caveolae‐like pits labeled with the black arrow, cytoplasmic membranous vesicles containing Fe‐C_3_N_4_ NSs labeled with the blue arrow, and membranous vesicles labeled with the red arrow. Relative viability of B16F10 cancer cells in d) Fe‐C_3_N_4_+H_2_O_2_, e) Fe‐C_3_N_4_+US, f) Fe‐C_3_N_4_ + H_2_O_2_ + US groups with different concentrations of Fe‐C_3_N_4_ NSs. Error bars were based on the standard deviations (SD) of five parallel samples. g) Relative viability of B16F10 cells after incubation with varied treatment regimens for 12 h. Error bars were based on the standard deviations (SD) of five parallel samples. The groups were: (G1) Control, (G2) US, (G3) C_3_N_4_, (G4) C_3_N_4_ + US, (G5) Fe‐C_3_N_4_, (G6) Fe‐C_3_N_4_ + US, (G7) Fe‐C_3_N_4_ + H_2_O_2_, (G8) Fe‐C_3_N_4_ + H_2_O_2_ + US. h) Fluorescent microscope images of B16F10 cells stained with Calcein‐AM (green, live cells) and PI (red, dead cells) after diverse treatments. Scale bar: 200 µm. i) Flow cytometry analysis of B16F10 cells stained with Annexin‐V FITC and PI after different treatments. j) Fluorescent microscope images of B16F10 cells stained with DCFH‐DA to detect ROS generation. Scale bar: 200 µm. k) Flow cytometry assay evaluating the ROS level of B16F10 cells after different treatments. (G1) Control, (G2) Fe‐C_3_N_4_, (G3) Fe‐C_3_N_4_ + H_2_O_2_, (G4) US, (G5) C_3_N_4_+US, (G6) Fe‐C_3_N_4_+US, (G7) Fe‐C_3_N_4_+ H_2_O_2_ +US.

The cellular internalization of as‐synthesized Fe‐C_3_N_4_ and C_3_N_4_ NSs by B16F10 mouse melanoma cells was assessed before exploring the antitumor effect induced by Fe‐C_3_N_4_ in vitro. Given that the C_3_N_4_‐based NSs could emit blue fluorescence under proper light excitation (Figure S12, Supporting Information),^[^ ^20a,c^ ^]^ we used fluorescence microscope to monitor the cellular uptake of C_3_N_4_ and Fe‐C_3_N_4_ NSs by B16F10 melanoma cells at various time points (1, 2, 4, and 6 h). The results proved that the cellular uptake of C_3_N_4_ and Fe‐C_3_N_4_ NSs increased in a time‐dependent manner (**Figure** **4**a and Figure S13, Supporting Information). Besides, the cellar uptake of C_3_N_4_ and Fe‐C_3_N_4_ NSs at 6 h was 96.35% and 97.29%, respectively, as measured by flow cytometry (Figure 4b). In addition, the endocytosis pathway of Fe‐C_3_N_4_ NSs entering B16F10 cells was via the caveolae‐mediated endocytosis based on biological electron microscopy. As shown in Figure 4c, the Fe‐C_3_N_4_ NSs were bonded to the B16F10 melanoma cell surface and moved to caveolae‐like pits, which were constructed from the cell membrane to form the cytoplasmic membranous vesicles containing Fe‐C_3_N_4_ NSs. Finally, Fe‐C_3_N_4_ NSs were released from the membranous vesicles into the cytoplasm to perform the specific function.

Thereafter, the in vitro cytotoxicity of Fe‐C_3_N_4_ and C_3_N_4_ NSs was evaluated by a typical cell‐counting kit 8 (CCK‐8) assay. As expected, the cell viability was higher than 90% even when the HUVEC cells were incubated with 80 µg mL^−1^ Fe‐C_3_N_4_ and C_3_N_4_ NSs for 48 h, demonstrating the favorable biosafety of Fe‐C_3_N_4_ and C_3_N_4_ NSs (Figures S14 and S15, Supporting Information). Notably, it was found that the viabilities of B16F10 cancer cells decreased to 65.19% after incubation with 80 µg mL^−1^ Fe‐C_3_N_4_ NSs for 48 h (Figure S16, Supporting Information), representing the distinct dose‐dependent and time‐dependent cytotoxicity of Fe‐C_3_N_4_ NSs toward cancer cells. The therapeutic efficacy on B16F10 cancer cells induced by synergistic SDT and CDT was further evaluated by the typical CCK‐8 assay. Considering that the concentration of H_2_O_2_ in TME was about 50–100 µm, the 100 µm H_2_O_2_ was selected for the following cellular experiments.^[^ ^4^, ^38^ ^]^ As predicted, the viabilities of B16F10 cancer cells decreased with the elevated Fe‐C_3_N_4_ NSs concentrations all in Fe‐C_3_N_4_ + H_2_O_2_ (CDT group), Fe‐C_3_N_4_ + US (SDT group) and Fe‐C_3_N_4_ + H_2_O_2_ + US (SDT + CDT group) (Figure 4d–g). Especially, the anticancer effect of SDT + CDT group was significantly higher than both CDT and SDT groups at the same concentration of Fe‐C_3_N_4_ NSs (*P* < 0.001) (Figure 4g), suggesting that the anticancer mechanism of the Fe‐C_3_N_4_ NSs was mainly dominated by synergistic SDT and CDT. The cell viability in the Fe‐C_3_N_4_ + US group was lower than C_3_N_4_ + US group (*P* < 0.001), further proving that Fe‐C_3_N_4_ NSs were an excellent chemoreactive sonosensitizer for augmenting SDT and endowing CDT effect to inhibit melanoma growth.

Furthermore, fluorescence microscope was used to observe the cellular state of B16F10 cancer cells after various treatments through double‐staining calcein acetoxymethyl ester (Calcein‐AM) with green fluorescence for live cells and propidium iodide (PI) with red fluorescence for dead cells. Compared with the US and C_3_N_4_ + US groups, the Fe‐C_3_N_4_ + US group exhibited higher red fluorescent intensity, illustrating that the Fe‐C_3_N_4_ NSs possessed an excellent SDT performance (Figure 4h). The best anti‐cancer effect was achieved in the Fe‐C_3_N_4_ + H_2_O_2_ + US group, and this outcome was consistent with the aforementioned CCK‐8 results, which further certified their bilateral enhancement of SDT and CDT activities. In addition, the flow cytometry assay based on the classical annexin V‐fluorescein isothiocyanate (FITC) and PI staining protocol was utilized to quantify the synergistic SDT and CDT performance of Fe‐C_3_N_4_ NSs against tumor cells. The apoptosis rates in various groups were consistent with the double‐staining calcein‐AM and PI assay (Figure 4h,i and Figure S17, Supporting Information).

It is well known that ROS is a key mediator for SDT and CDT to kill cancer cells, therefore the therapeutic efficiency of Fe‐C_3_N_4_ NSs depended on the intracellular ROS level to some extent. The typical probe 2′, 7′‐dichlorodihy drofluorescein diacetate (DCFH‐DA), which could be oxidized to green fluorescent 2, 7‐dichlorofluorescein (DCF) by ROS in cells, was used to detect the ROS generation after various therapies. As observed by fluorescence microscope, the Fe‐C_3_N_4_ + US group exhibited stronger green fluorescence in B16F10 cancer cells compared with both the US and C_3_N_4_ + US groups, indicating the superior SDT activity of the Fe‐C_3_N_4_ nanosystem (Figure 4j). Among different groups, the strongest green fluorescence was explicitly noticed in the Fe‐C_3_N_4_ + US + H_2_O_2_ group, indicating the large amounts of ROS generation induced by the bilateral enhancement of SDT and CDT based on Fe‐C_3_N_4_ NSs (Figure 4j). Furthermore, flow cytometry was performed to quantify the intracellular ROS generation, and the fluorescence intensity values under different treatments were consistent with the visual observation by fluorescence microscope (Figure 4k).

### 2.5 Transcriptome Analysis of Fe‐C_3_N_4_‐Mediated Combinatorial SDT and CDT

**Figure 5:**
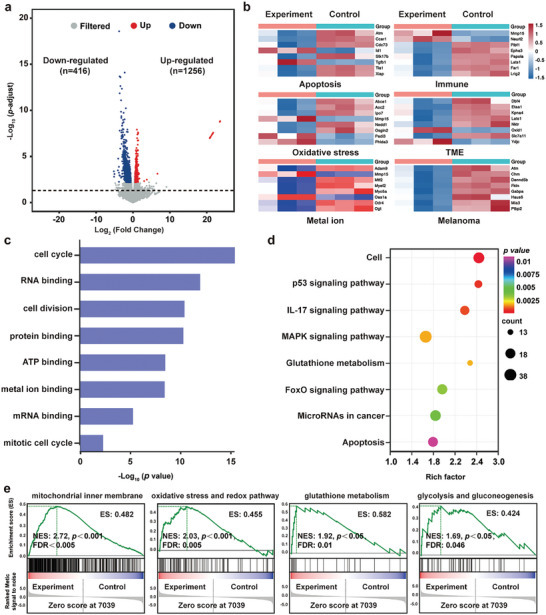
Biological mechanisms of bilateral enhancement of SDT and CDT by transcriptome analysis. a) Volcano plot in the Fe‐C_3_N_4_ + H_2_O_2_ + US (Experiment) group compared with the Control group (*P* < 0.05, |fold change| ≥ 1.5). b) Heat map of DEGs in the Experiment group as compared to the Control group. c) GO and d) KEGG enrichment analysis of differential signal pathways. e) GSEA enrichment plots of DEGs in the Experiment group compared with the Control group. B16F10 cells treated with Fe‐C_3_N_4_ + H_2_O_2_ + US as the Experiment group and without any treatment as the Control group.

For an in‐depth insight into the biological mechanism of Fe‐C_3_N_4_‐mediated combinatorial SDT and CDT for tumor therapy, RNAseq was used to analyze the gene expression in B16F10 cancer cells after being treated with Fe‐C_3_N_4_ + US + H_2_O_2_ (Experiment group) and without any remedy (Control group). As shown in **Figure** **5**a, a total of 1672 differentially expressed genes (DEGs) were ascertained with 416 up‐regulated genes (red) and 1256 down‐regulated genes (blue) in the Experiment group compared with the Control group (*P* < 0.05, |fold change| ≥ 1.5). From Figure 5b, the significant DEGs induced by the combinational SDT and CDT effect were mainly correlated with apoptosis, immune, oxidative stress, metal ion, TME, and melanoma. Especially, the abnormal expression of apoptosis‐related genes (Atm, Ccar1, Cdc73, *etc*.) suggested that the exogenous death receptor of B16F10 cancer cells was activated under the treatment of Fe‐C_3_N_4_ + H_2_O_2_ + US. In addition, DEGs (Abce1, Aoc2, Ipo7, etc.) involved in oxidative stress implied that ROS generation by synergistic SDT and CDT activities had intruded on the steady‐state and activated the oxidative stress system of B16F10 cancer cells.

Gene ontology (GO) enrichment analysis distinctly revealed that the DEGs after Fe‐C_3_N_4_‐mediated SDT and CDT treatment were mainly related to several biological processes and molecular functions of melanoma, such as cell cycle, ATP binding, metal ion binding, etc. (Figure 5c). The Kyoto encyclopedia of genes and genomes (KEGG) explicated that DEGs in the cell cycle, apoptosis, and IL‐17 signaling pathway promoted melanoma inhibition through regulating cell growth, apoptosis, and anti‐tumor immune responses (Figure 5d). Furthermore, gene set enrichment analysis (GSEA) was subjected to verify the therapeutic mechanism of synergetic SDT and CDT. It was found that mitochondrial inner membrane, oxidative stress and redox pathway, glutathione metabolism, and glycolysis and gluconeogenesis had significantly positive enrichment scores in the Experiment group (Figure 5e). Particularly, ROS‐induced oxidative stress was a strong driver of apoptosis through the enrichment of oxidative stress and the redox pathway (Figure 5e).

### 2.6 In Vivo Tumor Inhibition of Fe‐C_3_N_4_‐Based Thermogel under US Irradiation

First, the release profile of the Fe‐C_3_N_4_ NSs was investigated in simulated body fluid in vitro. As shown in Figure S18, Supporting Information, the cumulative release amount of Fe‐C_3_N_4_ NSs elevated in a time‐dependent manner. The release speed of Fe‐C_3_N_4_ NSs was fast in the early stage and slowed down gradually later. The cumulative release amount was about 52% at 24 h, 83% at 72 h, and 98% at 120 h. Then, we embedded Fe‐C_3_N_4_ NSs into an injectable thermogel (gel) precursor solution (defined as Fe‐C_3_N_4_‐Gel). This Gel with high biocompatibility and US stability is composed of chitosan (CTS) and *β*‐glycerophosphate (*β*‐GP),^[^ ^7^, ^39^ ^]^ which would achieve the solution‐Gel transformation and restrict the flow of Fe‐C_3_N_4_ NSs suspension after being injected into melanoma to further increase the residence time of Fe‐C_3_N_4_ NSs in tumor.^[^ ^7^, ^40^ ^]^ As shown in the SEM images and elemental mapping (Figures S19 and S20, Supporting Information), Fe‐C_3_N_4_‐Gel after lyophilization exhibited an irregular porous structural framework, and Fe atoms were scattered over the representative elements of CTS‐*β*‐GP Gel including sodium (Na), phosphorus (P), oxygen (O), carbon (C) and nitrogen (N). The blank Gel and Fe‐C_3_N_4_‐Gel could be transformed from liquid‐like status to the elastic Gel‐like matrix at 37 °C for 30 min, further proving that the Gel would realize phase transition at physiological temperature (Figure S21, Supporting Information).^[^ ^7^ ^]^

It is generally known that favorable biocompatibility and biosafety of biomaterials are crucial for promoting their applications in vivo. Herein, a series of experiments including the body weight, hematological analysis, and hematoxylin‐eosin (H&E) staining of main organs of mice were carried out to ensure safe bioapplication after subcutaneous injection with Fe‐C_3_N_4_‐Gel at various doses (Fe‐C_3_N_4_ NSs: 0.8, 1.6, and 3.2 mg kg^−1^). During the continuous monitoring of body weight for 14 days, no significant abnormal changes were observed in each group (Figure S22, Supporting Information), indicating the favorable biosafety of Fe‐C_3_N_4_‐Gel. The hematological variables, hepatic and renal functions were within the normal ranges in all groups (Figure S23, Supporting Information), demonstrating that the Fe‐C_3_N_4_‐Gel had no obvious hematologic, hepatic, or kidney toxicities. From the H&E staining images, histological analysis of major organs (including heart, liver, spleen, lung, and kidney) exhibited no apparent pathological abnormalities or inflammation in all mice (Figure S24, Supporting Information). These results confirmed the desirable biocompatibility and biosafety of Fe‐C_3_N_4_‐Gel and established a foundation for the follow‐up in vivo experiments.

**Figure 6:**
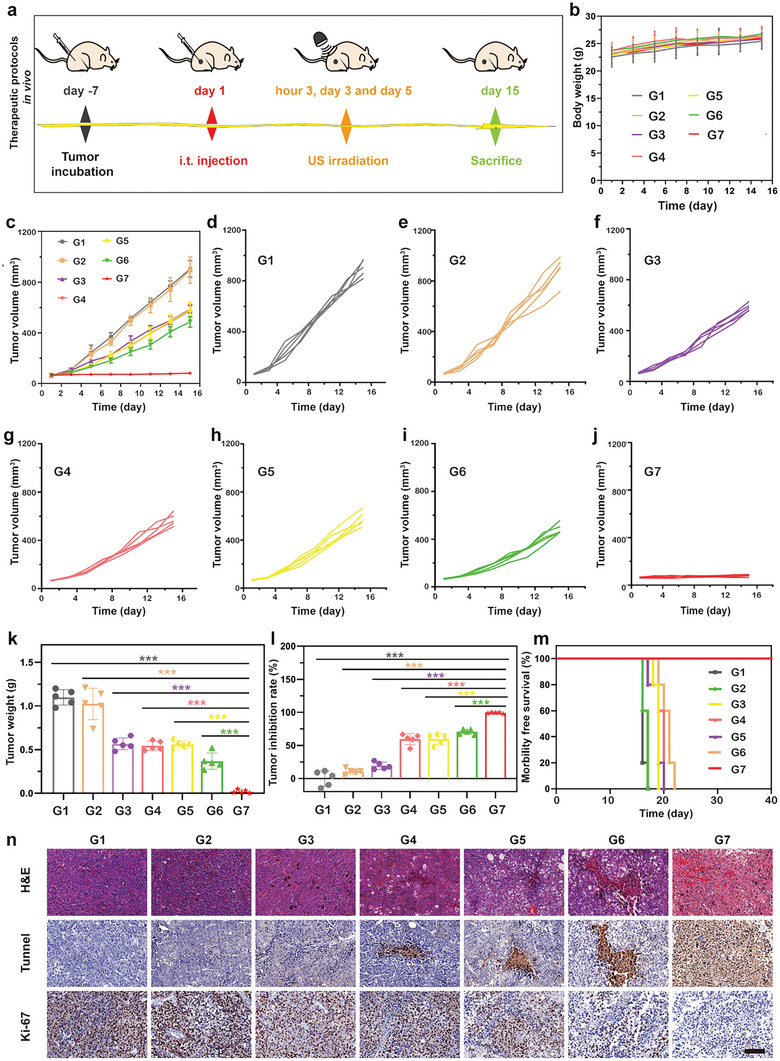
Synergistic enhancement of SDT and CDT for in vivo tumor inhibition. a) Therapeutic protocols in vivo. b) Time‐related bodyweight of mice in different treatment groups. (*n* = 5). c) Tumor volume growth curves in diverse treatment groups. (*n* = 5). d–j) Growth curve of tumor volume for each mouse in each group. (*n* = 5). k) Tumor weights of mice in all groups at the endpoint. l) Tumor inhibition rate of each group. Error bars were based on the standard deviations (SD) of five parallel samples. m) Survival curves of B16F10 tumor‐bearing mice after different treatments. (*n* = 5). n) H&E, TUNEL, and Ki‐67 staining in tumor tissues from various groups. Scale bar: 100 µm. (G1) Control, (G2) Gel, (G3) Fe‐C_3_N_4_‐Gel, (G4) US, (G5) Gel + US, (G6) C_3_N_4_‐Gel + US and (G7) Fe‐C_3_N_4_‐Gel + US.

Based on the excellent therapeutic efficiency of Fe‐C_3_N_4_ NSs in vitro and outstanding biocompatibility of Fe‐C_3_N_4_‐Gel in vivo, we further organized seven groups (Control, Gel, Fe‐C_3_N_4_‐Gel, US, Gel + US, C_3_N_4_‐Gel + US, and Fe‐C_3_N_4_‐Gel + US) to explore the in vivo tumor inhibition effect of Fe‐C_3_N_4_‐Gel upon US irradiation in the B16F10 xenograft melanoma model. US irradiation (1.5 W cm^−2^, 1.0 MHz, 50% duty cycle, 10 min) was conducted at 3 h, 3 days, and 5 days post intratumor injection of corresponding Gel (at the Fe‐C_3_N_4_/C_3_N_4_ NSs dose of 0.8 mg kg^−1^) (**Figure** **6**a). There was no significant difference in the body weight of mice among all groups during the continuous observation period, indicating that all the treatments would not cause acute toxicity at the experimental dose (Figure 6b). The photographs of B16F10 tumor‐bearing mice and tumors on day 15 (Figures S25 and S26, Supporting Information), and growth curves of tumor volumes (Figure 6c–j) and tumor weight (Figure 6k) manifested positive tumor restraint in the Fe‐C_3_N_4_‐Gel group and C_3_N_4_‐Gel + US group. In especial, the Fe‐C_3_N_4_‐Gel + US group with the tumor inhibition rate of as high as 98% was obviously higher than other groups (Figure 6l), corroborating the eminent anticancer efficacy of Fe‐C_3_N_4_‐triggered bilaterally enhanced SDT and CDT activities. The major organs (heart, liver, spleen, lung, and kidney) of all groups revealed no visible damage or inflammatory lesions in the histological sections stained with H&E, indicating the neglectable side effects after these therapeutic procedures in vivo (Figure S27, Supporting Information). It is worth noting that the lifespans of mice in the Fe‐C_3_N_4_‐Gel + US group were longer over 40 days, whereas those of the other groups did not exceed 22 days (Figure 6m), further proving the excellent cancer‐combating performance and biocompatibility of the synergic treatment strategy of SDT and CDT mediated by Fe‐C_3_N_4_ NSs. Ultimately, the exhaustive histological analysis of H&E, TdT‐mediated dUTP nickend labeling (TUNEL), and Ki‐67 further evaluated the therapeutic effect and mechanism of Fe‐C_3_N_4_‐based synergistic SDT and CDT (Figure 6n). Compared to the other groups, Fe‐C_3_N_4_‐Gel + US group exhibited the most severe tumor necrosis in the H&E staining, and the highest proportion of cell apoptosis in the TUNEL assay. In addition, the minimum amount of Ki‐67 positive cells was observed by Ki‐67 staining in the Fe‐C_3_N_4_‐Gel + US group, which was consistent with the above H&E and TUNEL staining data. The aforementioned results suggested that Fe‐C_3_N_4_‐mediated combinatorial SDT and CDT could inhibit tumor growth effectively.

## 3 Conclusion

In summary, we have successfully proposed and demonstrated the distinctive Fe‐C_3_N_4_ chemoreactive nanosonosensitizers for efficient melanoma therapy, which were synthesized by incorporating single‐atom Fe onto C_3_N_4_‐based nanostructure. The engineered Fe‐C_3_N_4_ NSs served as a new class of inorganic nanosonosensitizers achieving high yields of ROS production for melanoma treatment, due to the improved separation efficiency of e^−^‐h^+^ pairs induced by Fe single atoms incorporation under US irradiation. Besides, benefiting from the single‐atom Fe doping, Fe‐C_3_N_4_ NSs could act as a POD‐like enzyme to catalyze the Fenton chemical reaction for transforming overexpressed H_2_O_2_ into ∙OH, which could be enhanced by and cooperated with SDT for augmented antitumor efficacy. The DFT simulation authenticated that the underlying mechanism of Fe‐C_3_N_4_‐mediated SDT and CDT activities was based on the significant charge redistribution induced by the doping of the Fe atom in C_3_N_4_ NSs. As verified both in vitro and in vivo, Fe‐C_3_N_4_ NSs manifested outstanding antitumor‐therapeutic effect via synergetic and enhanced sono‐chemodynamic effect, and its underlying mechanism was dependent on the regulation of oxidative stress response, ROS generation, and apoptosis confirmed by RNAseq. The construction of Fe‐C_3_N_4_ NSs provided an efficient single‐atom doping strategy for inorganic nanosonosensitizers to improve ROS production under US stimulus and developed a span‐new window for outstanding tumor inhibition applications of semiconductor‐based inorganic sonosensitizers.

## Conflict of Interest

The authors declare no conflict of interest.

## Supporting information

Supporting Information

## Data Availability

The data that support the findings of this study are available from the corresponding author upon reasonable request.
