# Supplementary material for: Single Atom Iron‐Doped Graphic‐Phase C3N4 Semiconductor Nanosheets for Augmented Sonodynamic Melanoma Therapy Synergy with Endowed Chemodynamic Effect
Source: Adv Sci (Weinh). 2023 Jun 6;10(23):2302579. doi: 10.1002/advs.202302579 (PMC10427360; doi:10.1002/advs.202302579)
Supplement: Supplementary file 1 — Supporting Information [file ADVS-10-2302579-s001.pdf]

ADVANCED SCIENCE
Open Access

Supporting Information

for *Adv. Sci.*, DOI 10.1002/advs.202302579

Single Atom Iron-Doped Graphic-Phase C_3N_4 Semiconductor Nanosheets for Augmented Sonodynamic Melanoma Therapy Synergy with Endowed Chemodynamic Effect

Guiying Feng, Hui Huang, Min Zhang, Zhuole Wu, Dandan Sun, Qiqing Chen, Dayan Yang, Yuanyi Zheng, Yu Chen* and Xiangxiang Jing**

ADVANCED SCIENCE
Open Access

Supporting Information

for *Adv. Sci.*, DOI 10.1002/advs.202302579

Single Atom Iron-Doped Graphic-Phase C_3N_4 Semiconductor Nanosheets for Augmented Sonodynamic Melanoma Therapy Synergy with Endowed Chemodynamic Effect

Guiying Feng, Hui Huang, Min Zhang, Zhuole Wu, Dandan Sun, Qiqing Chen, Dayan Yang, Yuanyi Zheng, Yu Chen* and Xiangxiang Jing**

Supporting Information

Single Atom Iron-Doped Graphic-Phase C_3N_4 Semiconductor Nanosheets for Augmented Sonodynamic Melanoma Therapy Synergy with Endowed Chemodynamic Effect

Guiying Feng#, Hui Huang#, Min Zhang#, Zhuole Wu, Dandan Sun, Qiqing Chen, Dayan Yang, Yuanyi Zheng*, Yu Chen*, and Xiangxiang Jing*

G. Feng, M. Zhang, Z. Wu, D. Sun, Q. Chen, D. Yang, Prof. X. Jing
Department of Ultrasonography, Hainan General Hospital/Hainan Affiliated Hospital of Hainan Medical University, Haikou 570311, P. R. China
E-mail: ljjxx@hainmc.edu.cn (X. Jing)

Prof. Y. Zheng
Department of Ultrasound in Medicine, Shanghai Jiao Tong University Affiliated Sixth People's Hospital, State Key Laboratory of Oncogenes and Related Genes, Shanghai Jiao Tong University School of Medicine, Shanghai 200032, P. R. China
E-mail: zhengyuanyi@sjtu.edu.cn

H. Huang, Prof. Y. Chen
Materdicine Lab, School of Life Sciences, Shanghai University, Shanghai 200444, P. R. China
Email: chenyuedu@shu.edu.cn (Y. Chen)

# Equal contribution author.

A: Experimental Section

Materials: Anhydrous ferric chloride ($FeCl_3$), melamine (MA), and absolute methanol (MeOH) were purchased from Aladdin Biochemical Technology Co., Ltd. (Shanghai, China), chitosan (CTS, deacetylation degree: 75-85 and viscosity: 0.2-0.8 Pa·s at 25^{-1}), β-Glycerophosphate (β-GP) were received from Sigma-Aldrich Co. Ltd. (St. Louis MO, USA). 5, 5-dimethyl-1-pyrroline N-oxide (DMPO), 2, 2, 6, 6-tetramethyl-4-piperidone hydrochloride (TEMP) were received from Dojindo Molecular Technologies, Inc. (Shanghai, China). Hydrogen peroxide (H_2O_2), hydrochloric acid and acetic acid (AC, 0.1 M) was obtained from China National Pharmaceutical Group Co. (Beijing, China). 1,3-diphenylisobenzofuran (DPBF), 3, 3', 5, 5'-tetramethylbenzidine dihydrochloride (TMB), typical cell-counting kit 8 (CCK-8), calcein acetoxymethyl ester (Calcein AM), and propidium iodide (PI), annexin V, FITC apoptosis detection kit, and reactive oxygen species assay kit were obtained from Solarbio science & technology Co. ltd. (Beijing, China). TRIzol was purchased from Absin Bioscience Co. (Shanghai, China), calcium-and magnesium-free Dulbecco's phosphate-buffered saline (PBS), penicillin-streptomycin (100×) were obtained from Life Technologies Co. (Los Angeles, USA), standard fetal bovine serum (FBS) was purchased from Haoyang Biological Manufacture Co., Ltd. (Tianjin, China). Deionized (D.I.) water was generated using a Millipore Milli-Q system (Billerica, MA, USA). All chemicals were used directly without further purification.

Synthesis of Fe-C_3N_4 nanosheets (NSs): The massive Fe-doped C_3N_4 (bulk Fe-C_3N_4) was first obtained via a modified one-step pyrolysis method.[1] Typically, 4 g of MA was dispersed into 100 mL of MeOH solution under continuous stirring, and 200 mg of $FeCl_3$ was also added to this suspension to form a mixture. Then, the mixture was stirred continuously at 60 °C until

the MeOH was completely removed, and the obtained solid was transferred in a 30 mL ceramic boat to place into a furnace for calcination. The calcination process was kept at 500 °C for 4 h with the protection of nitrogen to obtain the brown-yellow powder named as bulk Fe-C_3N_4.

Subsequently, Fe-C_3N_4 nanosheets (NSs) were acquired through ultrasonic exfoliation[2] and pickling. In detail, 100 mg of bulk Fe-C_3N_4 was dissolved in 100 mL D.I. water and continuously exfoliated in the ultrasonic probe sonicator for 9 h. The initial formed suspension was centrifuged at about 10000 rpm to remove the residual unexfoliated bulk Fe-C_3N_4, and the supernatant was dried and immersed in hydrochloric acid for 48 h to obtain a coffee-like production named as Fe-C_3N_4 NSs. The C_3N_4 NSs were obtained by a similar strategy but without the addition of $FeCl_3$.

Synthesis of injectable Fe-C_3N_4-thermalgel (Gel): Firstly, 100 mg of CTS was dissolved in 4 mL AC upon stirring continuously for 12 h at ordinary temperature and then stored at 4 °C. 600 mg of β-GP powder was diffused in 1 mL D.I. water, and then sterilized through 0.22 μm syringe filter, and after that chilled to 4 °C for 20 min. Subsequently, the β-GP solution was added into the CTS solution slowly with the ice bath and whipped mixture uniformly to receive CTS-β-GP Gel precursor.[3] Finally, Fe-C_3N_4 NSs/C_3N_4 NSs/D.I. water was mixed with the Gel precursor with a volume ratio of 1:9, and then the mixture was shaken with a vortex mixer for 2 min to get the injectable Fe-C_3N_4-Gel, C_3N_4 -Gel and Gel, respectively.

Characterization: Scanning electron microscopy (SEM) images and energy dispersive X-ray spectroscopy (EDS) images were acquired on Hitachi Regulus 8100 electron microscopy (Hitachi Ltd., Japan). High-angle annular dark field-scanning transmission electron microscopy (HAADF-STEM) images and energy dispersive X-ray spectroscopy (EDS) images were collected using the Thermo Fisher Scientific Themis Z electron microscope (Thermo Fisher Sci-

entific Inc., US). Transmission electron microscopy (TEM) images were taken on a JEOL JEM-F200 transmission electron microscope (JEOL Ltd., Japan). The samples for TEM analysis were prepared by dipping the carbon-coated copper grids into ethanol solutions the samples and drying them under ambient conditions. Biological electron microscope images were taken on a Hitachi HT7800 electron microscopy (Hitachi Ltd., Japan). The thickness of the samples was measured by atomic force microscopy (AFM) on Bruker Dimension Icon (Bruker, Scientific Technology Ltd., US). The dissolved iron concentration was detected by an inductively coupled plasma-atomic emission spectrometer (ICP-AES) Agilent 7800 (Agilent Technologies Ltd., US). The structures of the materials were confirmed by X-ray powder diffraction (XRD) analysis on a Rigaku Ultima IV diffractometer (Cu Kα, λ = 1.5418 nm) (Rigaku Co., Japan) with a scanning angle ranging from 5 ° to 90 ° of 2θ. The surface chemical composition and the binding information of required elements were characterized by the X-ray photoelectron spectroscopy (XPS) technique on a Thermo Scientific K-Alpha system (Thermo Fisher Scientific Inc., US). The electron spin resonance (ESR) spectrum characterization was performed on a Bruker A5000 EMX electron paramagnetic resonance spectrometer (Bruker, Scientific Technology Ltd., US). Ultraviolet-visible diffuse reflection spectra (UV-vis DRS) were performed on a UV-3600i Plus spectrophotometer (Shimadzu Co., Japan) with the wavelength ranging from 200 nm to 800 nm by using BaSO4 powder as the reference. Fluorescence measurements were performed on a Edinburgh FLS1000 spectrometer (Edinburgh Instruments, England). Photoelectrochemical tests were conducted on a CHI760E electrochemistry workstation (Chenhua, Shanghai, China) with Ag/AgCl as the reference electrode and the counter electrode. Fluorescence microscope images were recorded by the Nikon Eclipse Ts2-FL (Nikon Co., Japan). Cell phagocytosis, apoptosis, and ROS were obtained by FACS Calibur flow cytometry (Becton, Dickinson, and Co., USA). Ultrasound (US) irradiation for sonodynamic therapy was conducted by an Intelect Transport Ultrasound (Well.d Medical Electronics Ltd., China).

Singlet oxygen ($^1O_2$) generation: First of all, TEMP was used as a radical spin trap for $^1O_2$ detected by ESR spectra. The five groups included C_3N_4, Fe-C_3N_4, US, C_3N_4 + US, and Fe-C_3N_4 + US groups were set to explore and compare the production of $^1O_2$, and the concentrations of C_3N_4 and Fe-C_3N_4 NSs were 80 μg mL^{-1}. 5 μL of TEMP (100 mM) was added into 100 μL of the Fe-C_3N_4/C_3N_4 solution and then irradiated by US (1.5 W cm^{-2}, 1.0 MHz, 50% duty cycle) for 2 min. In the end, 2 μL of the reaction mixture was injected into the quartz capillary and measured by the ESR spectrum immediately.

Moreover, DPBF was typically used as a molecular probe to detect $^1O_2$ generation. Fe-C_3N_4 NSs (80 μg mL^{-1}) were mixed with 40 μL DPBF (1 mg mL^{-1}) and then irradiated by US (1.5 W cm^{-2}, 1.0 MHz, 50% duty cycle) for different durations in the dark (0, 1, 2, 3, 4 and 5 min). In the course of time, the absorption intensity change of DPBF at 423 nm was recorded by a Tecan Spark microplate reader (Tecan, M. nnedorf, Switzerland). For comparison, the ROS generation of pure DI-Water and C_3N_4 triggered by US respectively acted as US group and C_3N_4 + US group were also detected in the same way.

Hydroxy radical (·OH) generation: DMPO was used as a radical spin trap for ·OH observed by ESR spectra. Typically, Fe-C_3N_4 NSs (80 μg mL^{-1}) and H_2O_2 (100 μM) were mixed with 10 μL DMPO (100 mM) solution in the mildly acidic environment (pH 5.4) with or without US irradiation and then detected by ESR spectra at once. Moreover, H_2O_2 with or without US irradiation and C_3N_4 NSs mixed with H_2O_2 were also performed with a similar approach for comparison.

In addition, TMB was used as a typical molecular probe to detect the ·OH production. Firstly, Fe-C_3N_4 NSs (80 μg mL^{-1}) were mixed with varying concentrations of H_2O_2 (100 μM, 1 mM and 10 mM) and 5 μL TMB (0.1 mg mL^{-1}) buffered at pH = 5.4 for 0, 1, 2, 3, 4 and 5

min. Subsequently, Fe-C_3N_4 NSs (80 μg mL^{-1}) were mixed with H_2O_2 (100 μM) and 5 μL TMB (0.1 mg mL^{-1}) at pH 5.4, and then the mixture was further treated with or without US (1.5 W cm^{-2}, 1.0 MHz, 50% duty cycle) in the dark for 0, 1, 2, 3, 4 and 5 min. After the reaction of above mixture, the absorption change of TMB at 650 nm was recorded by the microplate reader. Besides, H_2O_2 under US activation was performed in the same way for comparison.

Density functional theory calculations: All the geometry optimization and energy calculations were performed by using density functional theory (DFT) implemented in the Vienna ab initio simulation package (VASP).[4] The electron and core interactions were described using the frozen-core projected augmented wave (PAW) approach. Generalized gradient approximation (GGA) formulated by Perdew-Burke-Ernzerhof (PBE) was chosen for the exchange-correlation between electrons.[5] A kinetic energy cutoff of 400 eV was used for the plane wave. The Brilliouin zone was sampled using 5×5×2 k-point Gamma mesh for the orthogonal supercell of g-C_3N_4 containing 6 C and 8 N atoms to ensure adequate convergence. The van der Walls interaction was accounted for with the Grimme DFT-D3 correction.[6] The related structures were optimized until the energy differences converged within 10^{-4} eV and the forces of all atoms were less than 0.01 eV/Å.

Cellular uptake in vitro: B16F10 mouse melanoma cells were seeded in a 6-well plate at 2×10^5 cells in the logarithmic growth phase for 12 h. To discard the old medium after adhered plate of cells, and then added 2 ml Fe-C_3N_4/C_3N_4 NSs (40 μg mL^{-1}) which were dissolved in 1640 medium to the plate for different incubation times (1, 2, 4, 6 h). In the course of time, the cellular uptake of the NSs was observed by fluorescence microscope and biological electron microscope.

In addition, after the B16F10 cancer cells were incubated with Fe-C_3N_4/C_3N_4 NSs for 6 h, flow cytometry was employed to quantitatively analyze the fluorescence signal intensity of the cellular uptake of the NSs.

Cytotoxicity assay in vitro: CCK-8 assay was used to evaluate relative viability for HUVEC cells incubated with Fe-C_3N_4 and C_3N_4 NSs. HUVEC cells were cultivated in a 96-well plate at 1×10^4 cells in the logarithmic growth phase for 12 h, and then added different concentrations of Fe-C_3N_4/C_3N_4 NSs (0, 2.5, 5, 10, 20, 40 and 80 μg mL^{-1}) to substitute for old medium for 12 h, 24 h, and 48 h, respectively. At the end of co-incubation, 10 μL CCK-8 was added to the plate and further cultivated for 1 h, and the microplate reader was used to estimate the cell viability at 450 nm. Furthermore, the same CCK-8 method was used to examine the viability of B16F10 cancer cells in the following groups including Fe-C_3N_4, Fe-C_3N_4 + US, Fe-C_3N_4 + H_2O_2, and Fe-C_3N_4 + H_2O_2 + US groups with different concentrations (0, 2.5, 5, 10, 20, 40 and 80 μg mL^{-1} of Fe-C_3N_4 NSs). After further 6 h cultivation, the cells were irradiated with or without US and were incubated for another 6 h, 18 h, and 42 h, respectively. Additionally, the same CCK-8 assay was applied to compare the B16F10 cellular cytotoxicity of Control, US, C_3N_4, C_3N_4 + US, Fe-C_3N_4, Fe-C_3N_4 + US, Fe-C_3N_4 + H_2O_2 and Fe-C_3N_4 + H_2O_2 + US groups with the same concentration (40 μg mL^{-1}) of the NSs for 12 h (after further 6 h incubation, the cells were treated with or without US irradiation were incubated for another 6 h). The concentration of H_2O_2 was 100 μM, and the US irradiation time was 1 min (1.5 W cm^{-2}, 1.0 MHz, 50% duty cycle).

Live and dead cell staining assay: To further prove the cellular death induced by Fe-C_3N_4-mediated SDT and CDT, B16F10 cancer cells were divided into seven groups, including Con-

trol, Fe-C_3N_4, Fe-C_3N_4 + H_2O_2, US, C_3N_4 + US, Fe-C_3N_4 + US and Fe-C_3N_4 + H_2O_2 + US (concentrations of the NSs, H_2O_2 and the set of US were the same as mentioned above). According to the different groups, B16F10 cancer cells were co-incubated with corresponding NSs and H_2O_2 in a 6-well plate for 6 h, and then further cultivated for another 6 h after US irradiation or without US incited. Thereafter, the as-treated cells were stained with Calcein-AM (green color for live cells, 5 μM) and PI (red color for dead cells, 10 μM) in accordance with the instructions, and then observed by fluorescent microscope at once.

Cell apoptosis analyzed by flow cytometry: Flow cytometry was further used to estimate the B16F10 cancer cells apoptosis caused by the collaborative SDT and CDT effect. In brief, the B16F10 cells were remedied by the above seven groups and then collected to stain with 5 μL Annexin V-FITC and 10 μL PI for 15 min according to the illustrations. At last, the apoptosis of the treated cells was detected by flow cytometry.

Intracellular ROS generation: Both fluorescence microscope observation and flow cytometry were used to investigate intracellular ROS generation. B16F10 cancer cells were co-incubated in a 6-well plate with corresponding processes based on the above seven groups and then stained with 500 μL 2', 7'-dichlorofluorescin diacetate (DCFH-DA, 10 μM) on the basis of the prospectus. Ultimately, the stained cells were washed with PBS gently and then surveyed by fluorescence microscope immediately. Moreover, after the same remedy and staining of B16F10 cancer cells, flow cytometry was applied to gauge the fluorescence intensity of intracellular DCFH-DA.

Genome and transcriptome analyses: B16F10 cells were divided into two groups as Experi-

ment group with treatment by Fe-C_3N_4 + US + H_2O_2 and Control group without any remedy. After extracting total RNA with TRIzol, the RNA samples were sent to OE Biotech Co., Ltd. (Shanghai, China) for RNA sequencing. The gene expression data were analyzed by htseq-count and FPKM.[7] Furthermore, the emblematical of differentially expressed genes were dissected in the light of the Gene ontology (GO) and Kyoto encyclopedia of genes and genomes (KEGG) databases.[8]

Biocompatibility and biosafety in vivo: All animal experiments were performed with the approval of the ethics by the Ethics Committee of Shanghai University. ICR mice were divided into four groups (n = 5 per group), including (1) Control (subcutaneous injection with 50 μL of saline), (2) 0.8 mg kg^{-1} (subcutaneous injection with 50 μL of Fe-C_3N_4-Gel at the Fe-C_3N_4/C_3N_4 dose of 0.8 mg kg^{-1}), (3) 1.6 mg kg^{-1} (subcutaneous injection with 50 μL of Fe-C_3N_4-Gel at the Fe-C_3N_4/C_3N_4 dose of 1.6 mg kg^{-1}), and (4) 3.2 mg kg^{-1} (subcutaneous injection with 50 μL of Fe-C_3N_4-Gel at the Fe-C_3N_4/C_3N_4 NSs dose of 3.2 mg kg^{-1}) to explore biocompatibility and biosafety *in vivo*. The body weight of each mouse was recorded every two days until the 15th day, and the blood was collected at day 15 after injection for routine blood and serum biochemical analysis. Furthermore, the main organs of one mouse from each group were randomly selected for hematoxylin-eosin (H&E) histological analysis.

Therapeutic effects in vivo: First of all, B16F10 cells (1×10^6) were suspended in 100 μL of PBS, and then subcutaneously injected into the right back of male BALB/c nude mice (5-7 weeks, ~23 g) to establish the tumor model. As the tumor volume reached around 60-70 mm^3, the mice were randomly divided into seven groups (non-treated group as Control, Gel, Fe-C_3N_4-Gel, US, Gel + US, C_3N_4-Gel + US, and Fe-C_3N_4-Gel + US, n = 5 per group). The mice

in all groups were intratumorally injected with 50 μL corresponding Gel/C_3N_4-Gel/Fe-C_3N_4-Gel at the Fe-C_3N_4/C_3N_4 dose of 0.8 mg kg^{-1} except for the Control and US groups, and then irradiated by US (1.5 W cm^{-2}, 1.0 MHz, 50% duty cycle, 10 min) at 3 hours, 3 days and 5 days after the injection. The body weight and tumor volume (volume = (length × $width^2$)/2) were measured every two days, and the tumor-bearing mice were photographed on day 1, day 7, and day 15 until the 15th day. The mice should be euthanized as tumors were larger than 1000 mm^3 in accordance with the standard animal protocol, and the tumors were dissected to weigh and photograph for evidence. At the end of the various treatments, the blood of mice was collected for routine blood and serum biochemical analysis, and the tumors were taken for H&E, TdT-mediated dUTP nickend labeling (TUNEL), and Ki-67 for exhaustive histological analyses. In addition, the major organs of one mouse from each group were randomly collected for H&E histological analysis.

Statistical analysis: All quantitative values were presented as mean ± standard deviation (SD). The statistical significance of differences among groups was carried out by using one-way analysis of variance analysis (ANOVA) followed by Tukey's post-test. The statistical significance for the tests was at $*P < 0.05$, $**P < 0.01$ and $***P < 0.001$, NS indicates $P > 0.05$. Statistical product service solutions 23 (International Business Machines Co., NY, USA) was used for all statistical analyses. Flow cytometry data were visualized and analyzed by using FlowJo (v10.6.2).

B: Supplementary figures

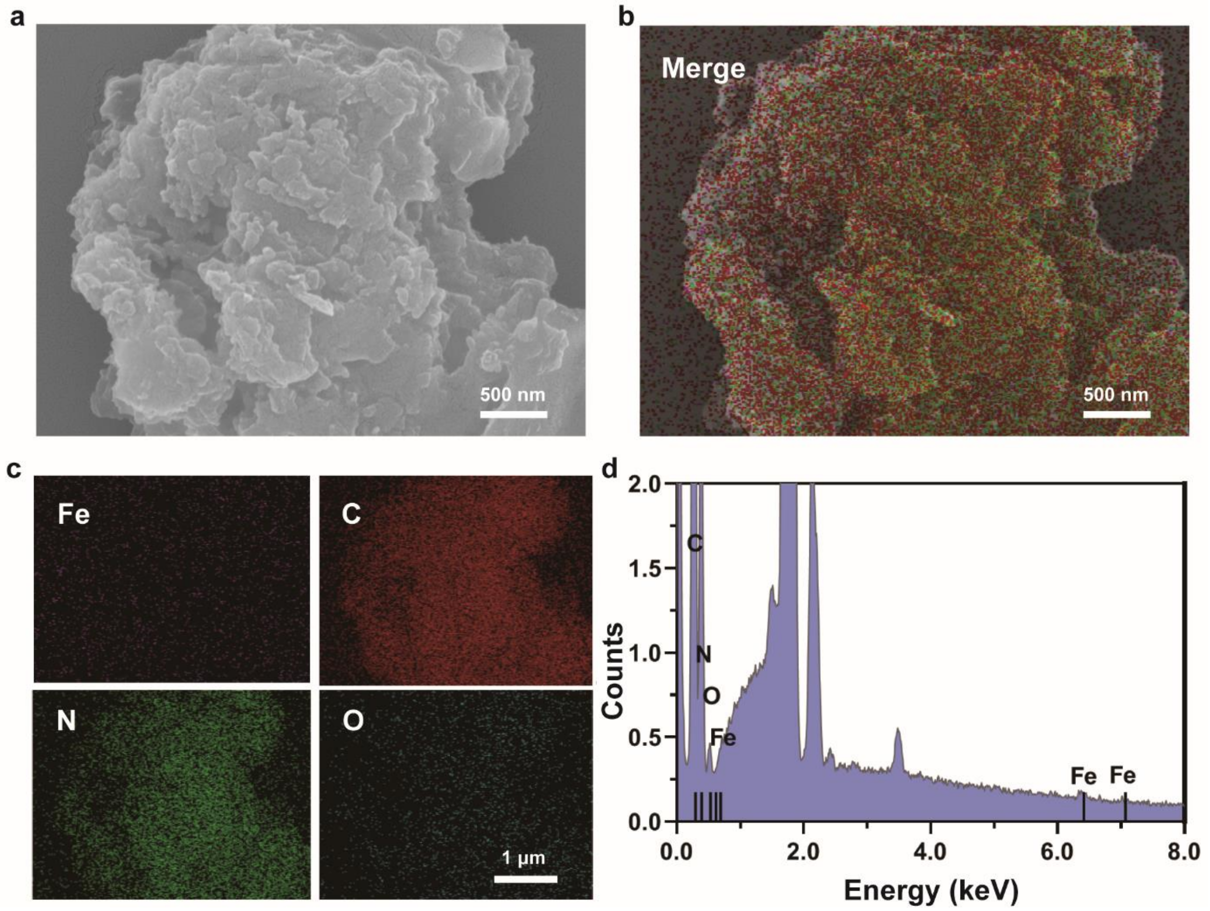

Figure S1. Characterization of bulk Fe-C_3N_4. (a) SEM image, (b-c) the corresponding elemental mapping of Fe, C, and N, and (d) EDS of bulk Fe-C_3N_4.

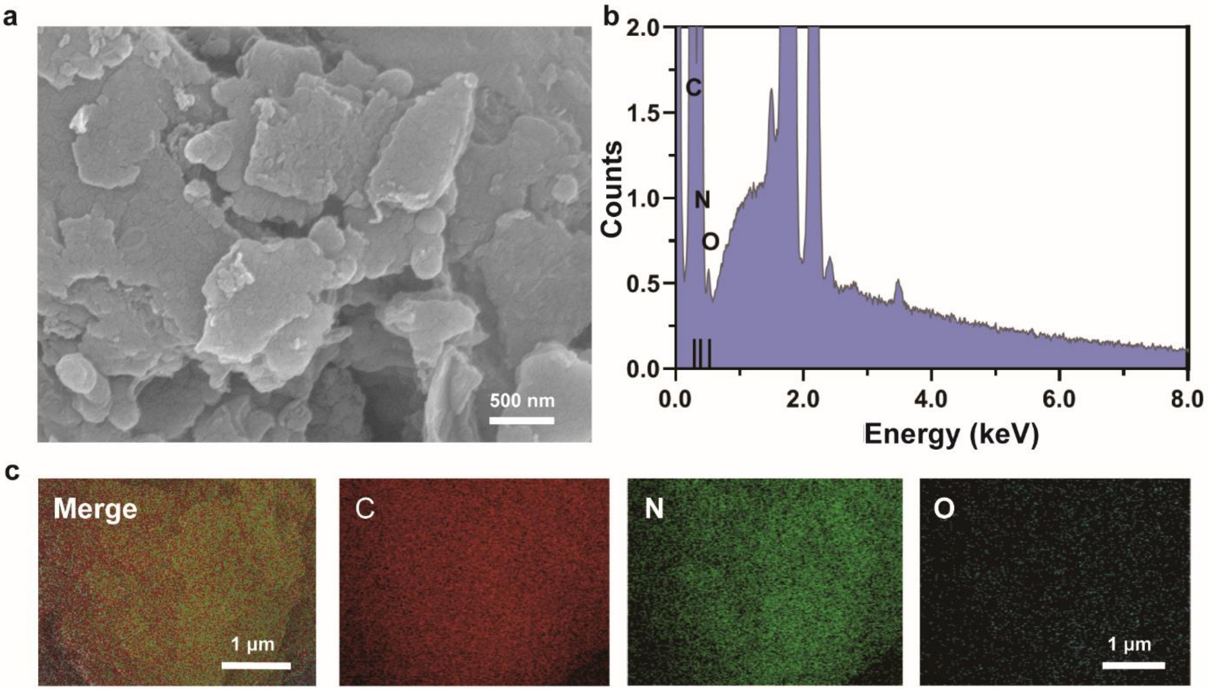

Figure S2. Characterization of bulk C_3N_4. (a) SEM image, (b) EDS, and (c) the corresponding elemental mapping of C, N, and O of bulk C_3N_4.

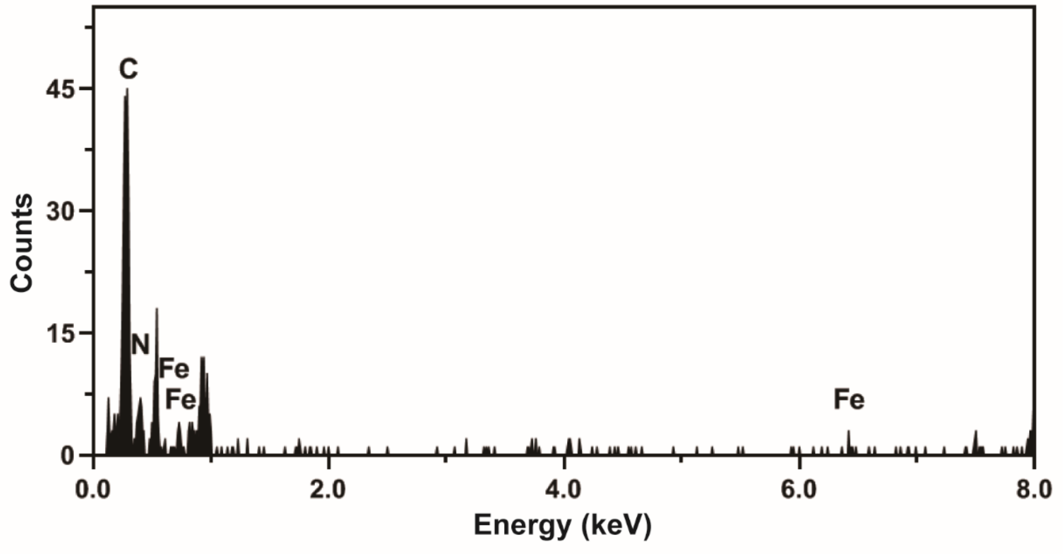

Figure S3. EDS of Fe-C_3N_4 NSs corresponding to the HAADF-STEM image.

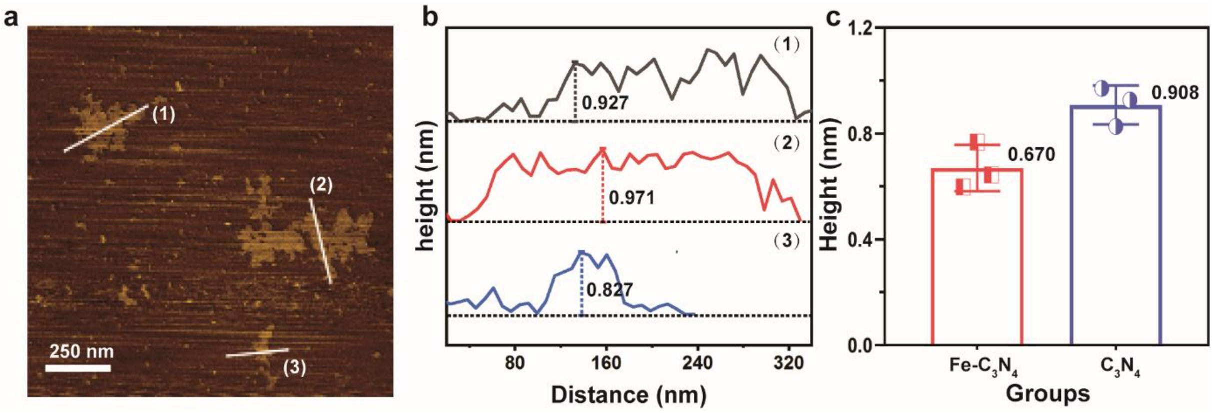

Figure S4. (a-b) AFM image and the corresponding height of C_3N_4 NSs. (c) The average height of Fe-C_3N_4 and C_3N_4 NSs.

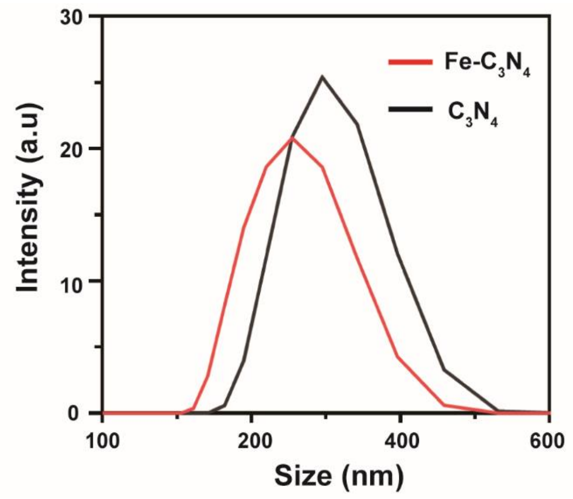

Figure S5. Hydrodynamic diameter of Fe-C_3N_4 and C_3N_4 NSs measured by DLS.

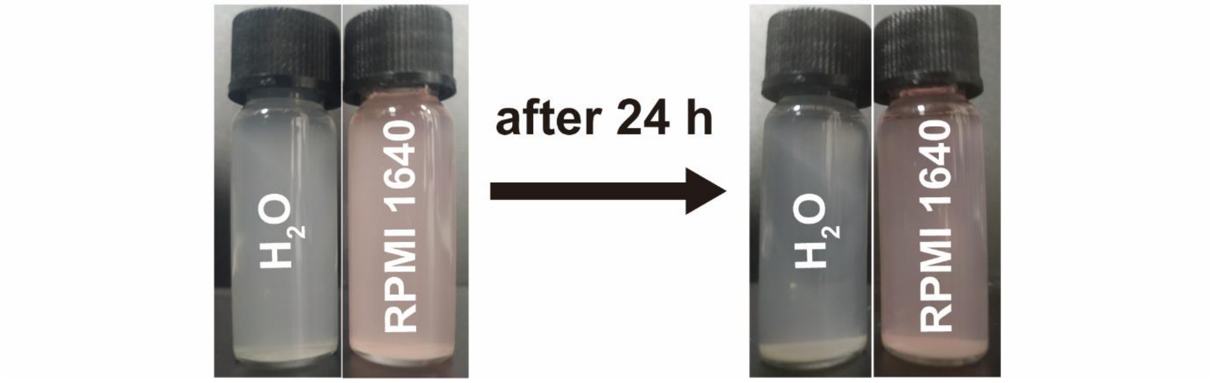

Figure S6. The digital photographs of the Fe-C_3N_4 NSs dispersed in water and RPMI 1640 for 24 h.

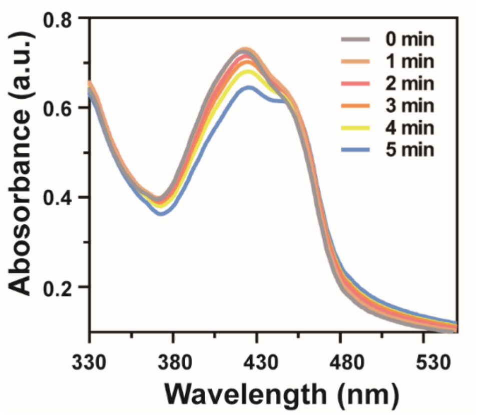

Figure S7. The absorption intensities of DPBF treated with DI-water followed by US irradiation for different durations (0, 1, 2, 3, 4, and 5 min).

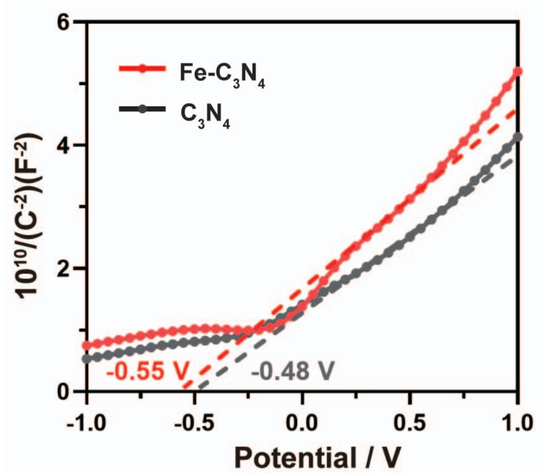

Figure S8. Mott–Schottky plots of Fe-C_3N_4 and C_3N_4 NSs.

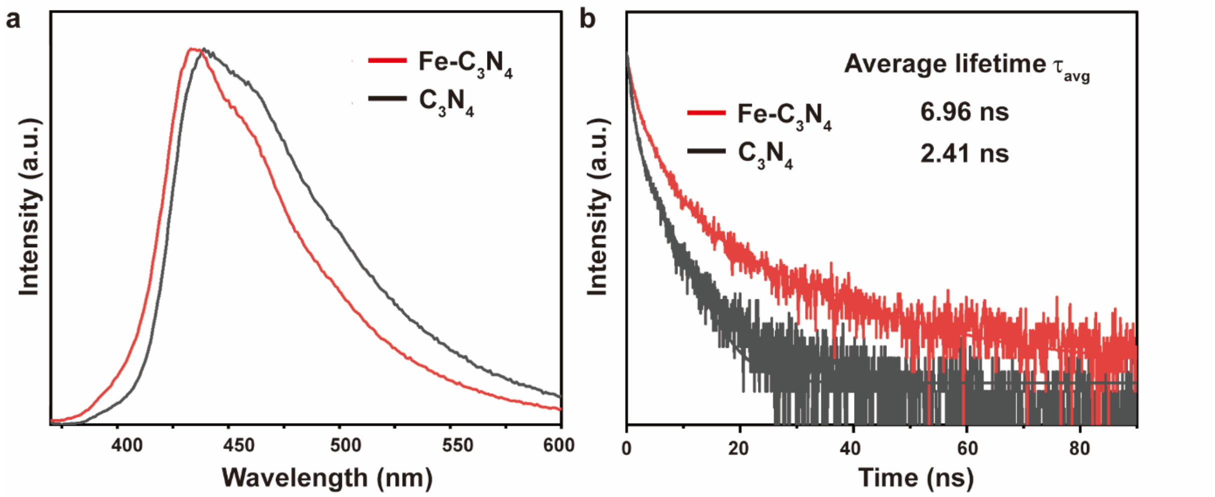

Figure S9. (a) The steady-state fluorescent spectra and (b) time-resolved fluorescent spectra of Fe-C_3N_4 and C_3N_4 NSs.

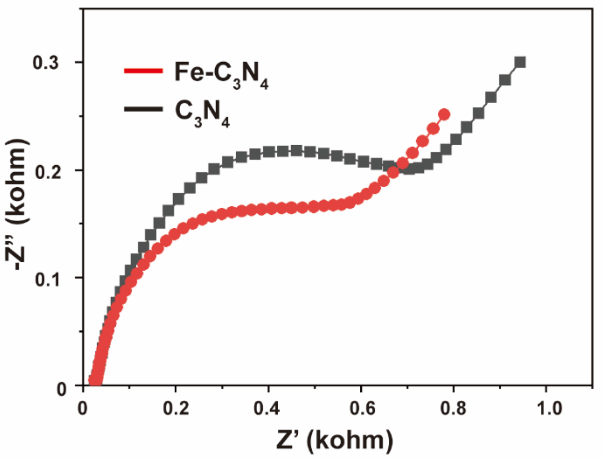

Figure S10. The electrochemical impedance spectroscopy of Fe-C_3N_4 and C_3N_4 NSs.

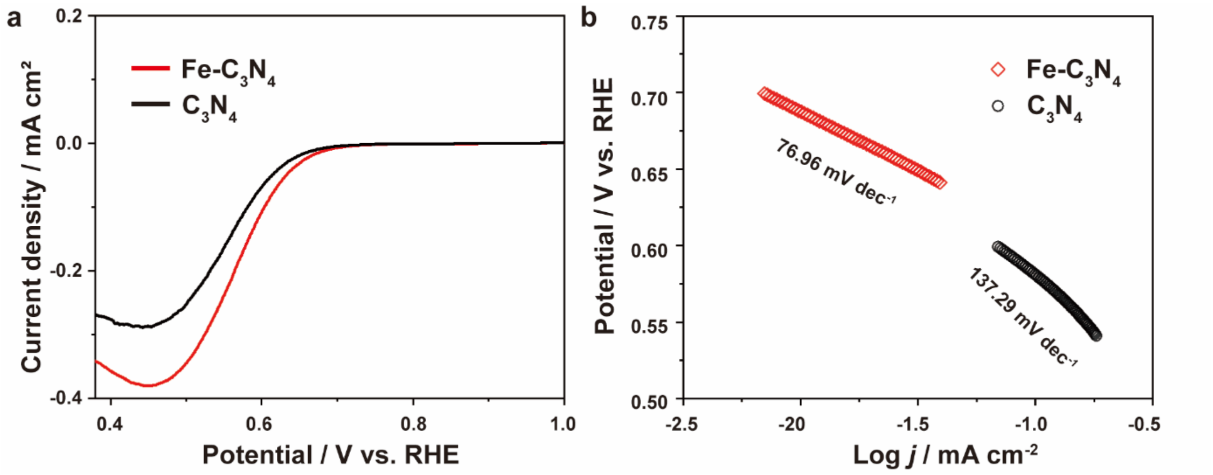

Figure S11. (a) The linear sweep voltammetry (LSV) curves of Fe-C_3N_4 and C_3N_4 NSs. (b) Tafel slope plots of Fe-C_3N_4 and C_3N_4 NSs derived from the LSV results.

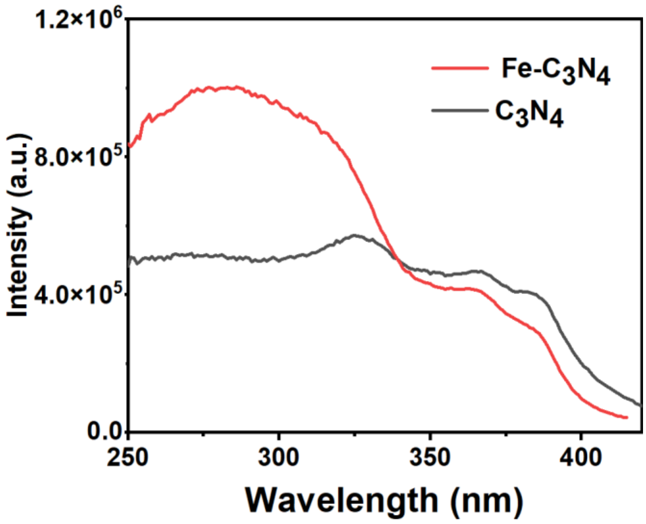

Figure S12. The light excitation wavelength of blue fluorescence emitted by C_3N_4 and Fe-C_3N_4.

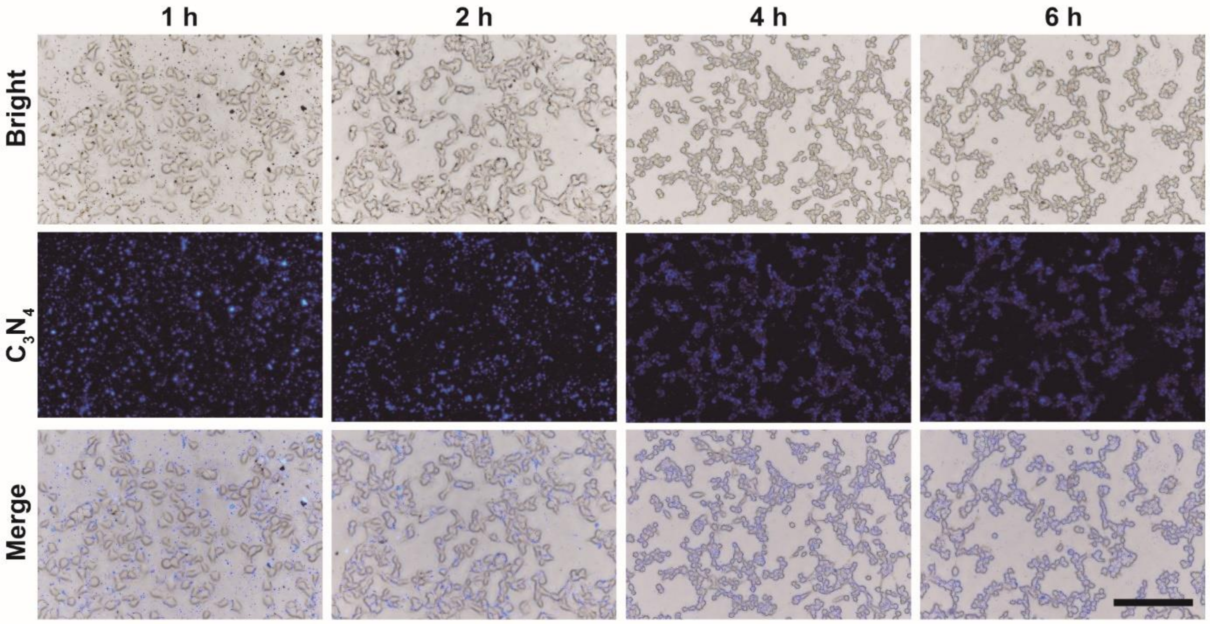

Figure S13. The cellular uptake of C_3N_4 NSs at 1, 2, 4, and 6 h. (Scale bar: 200 μm).

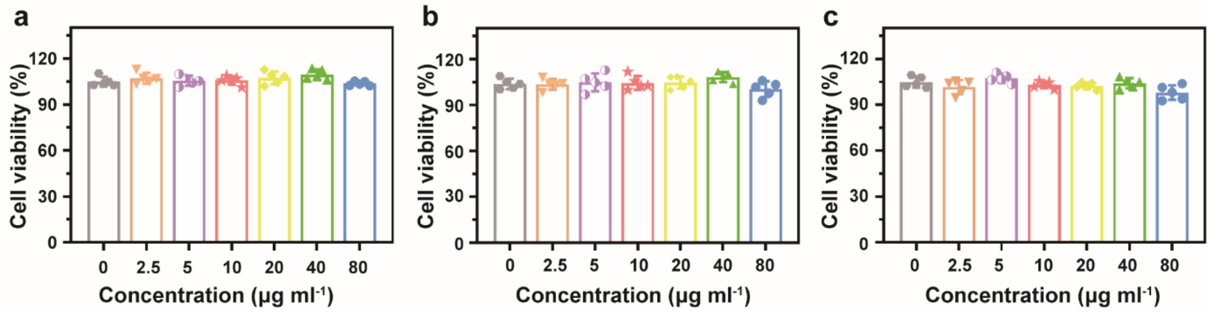

Figure S14. (a-c) Relative viability of HUVEC cells after incubation with elevated concentrations of Fe-C_3N_4 NSs (0, 2.5, 5, 10, 20, 40, and 80 μg mL^{-1}) for (a) 12 h, (b) 24 h, and (c) 48 h. Error bars were based on the standard deviations (SD) of five parallel samples.

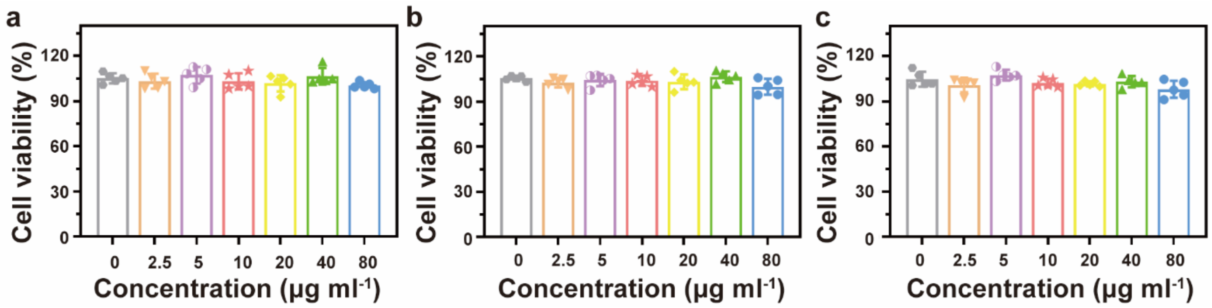

Figure S15. (a-c) Relative viability of HUVEC cells after incubation with elevated concentrations of C_3N_4 NSs (0, 2.5, 5, 10, 20, 40, and 80 μg mL^{-1}) for (a) 12 h, (b) 24 h, and (c) 48 h. Error bars were based on the standard deviations (SD) of five parallel samples.

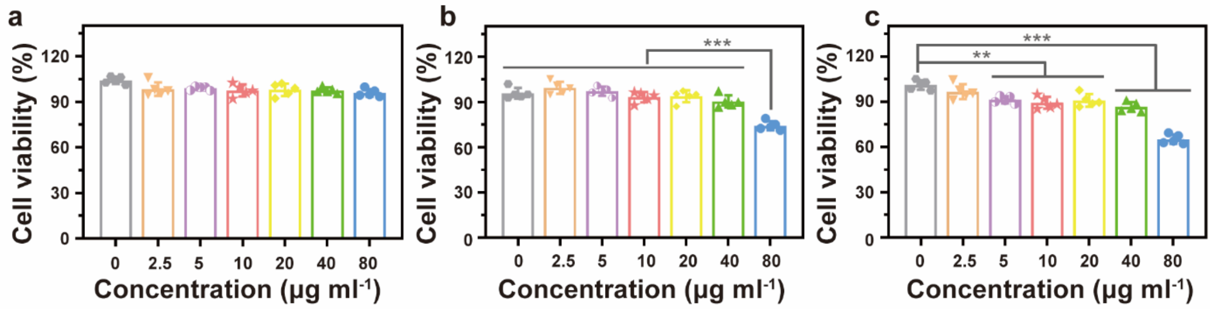

Figure S16. (a-c) Relative viability of B16F10 cancer cells after incubation with elevated concentrations of Fe-C_3N_4 NSs (0, 2.5, 5, 10, 20, 40, and 80 μg mL^{-1}) for (a) 12 h, (b) 24 h, and (c) 48 h. Error bars were based on the standard deviations (SD) of five parallel samples.

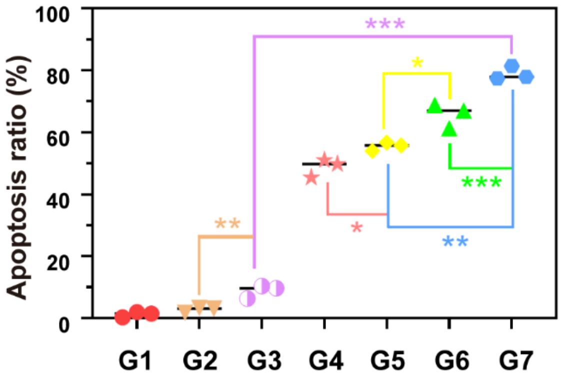

Figure S17. The apoptosis ratio of B16F10 cancer cells after various treatments. Error bars were based on the standard deviations (SD) of three parallel samples. The groups were: (G1) Control, (G2) Fe-C_3N_4, (G3) Fe-C_3N_4+ H_2O_2, (G4) US, (G5) C_3N_4+US, (G6) Fe-C_3N_4+US, (G7) Fe-C_3N_4+ H_2O_2 +US.

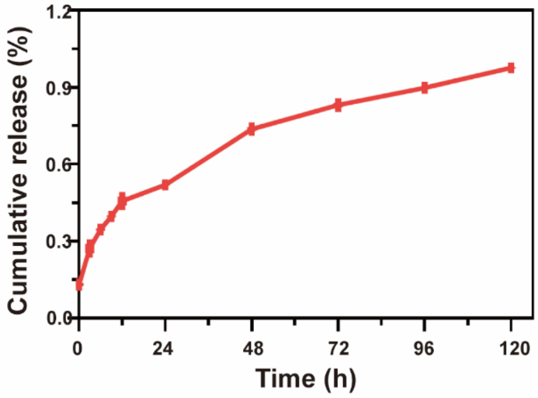

Figure S18. Release kinetic of Fe-C_3N_4 NSs.

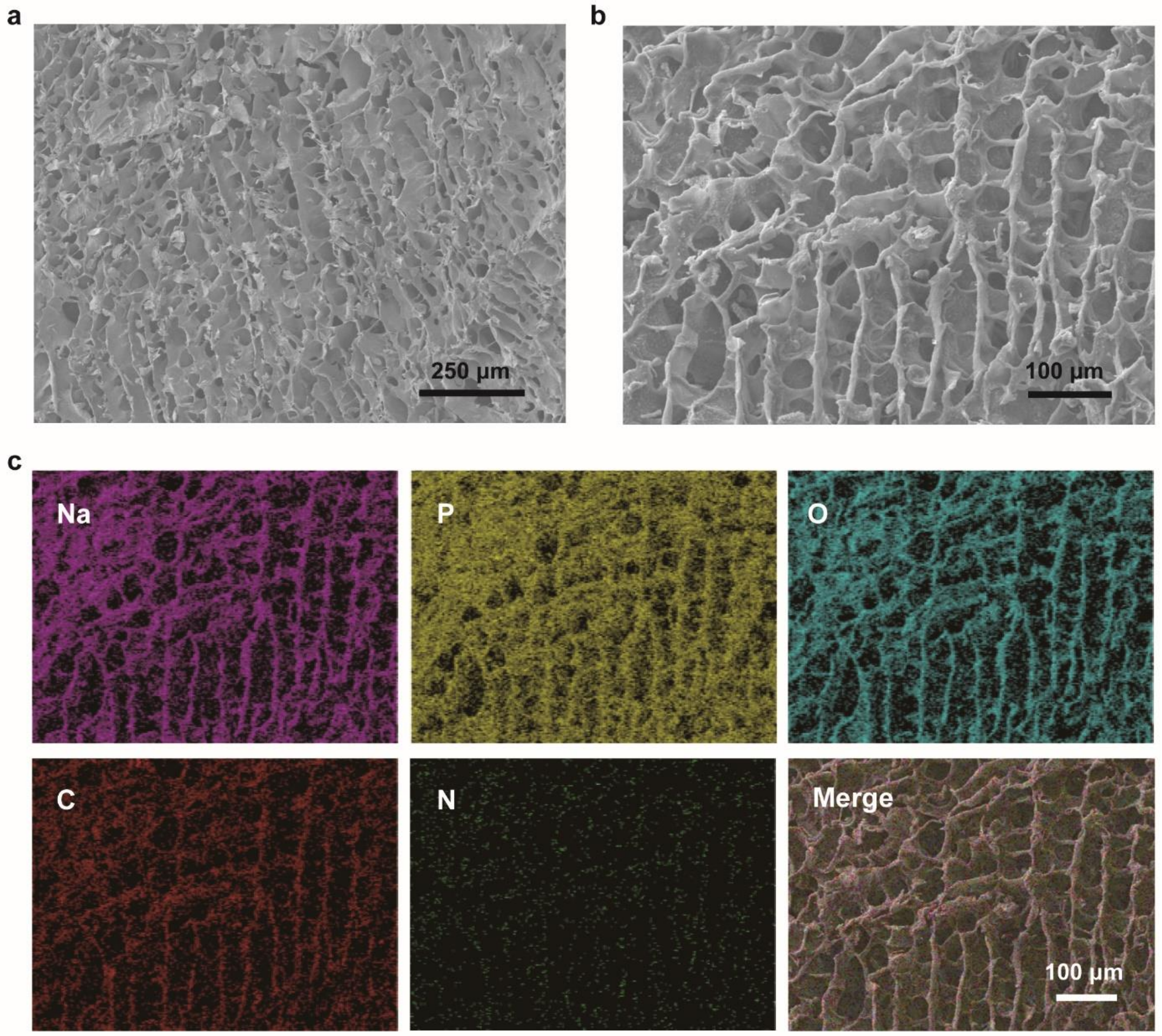

Figure S19. SEM image of Gel. (a-b) SEM images and (c) corresponding elemental mapping images of Gel.

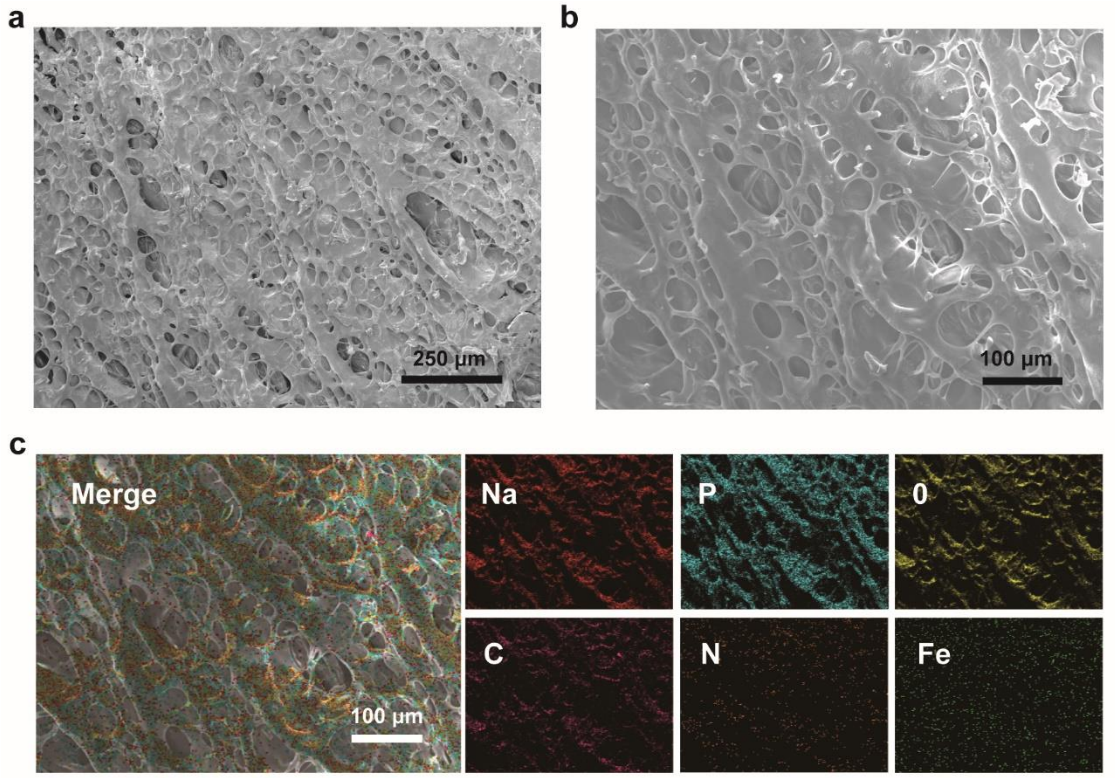

Figure S20. SEM image of Fe-C_3N_4-Gel. (a-b) SEM images and (c) corresponding elemental mapping images of Fe-C_3N_4-Gel.

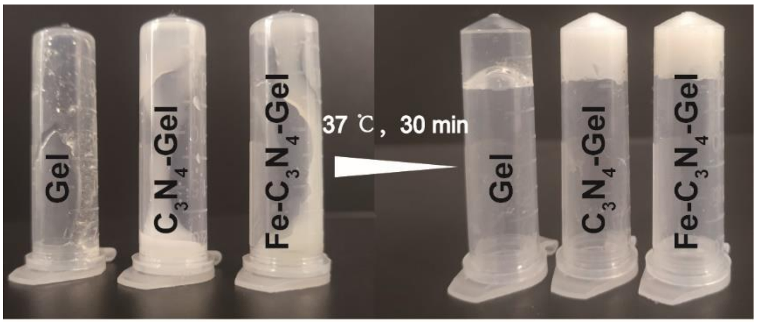

Figure S21. Morphology of the Gel, C_3N_4-Gel, and Fe-C_3N_4-Gel before and after water bathing for 30 min at 37 °C.

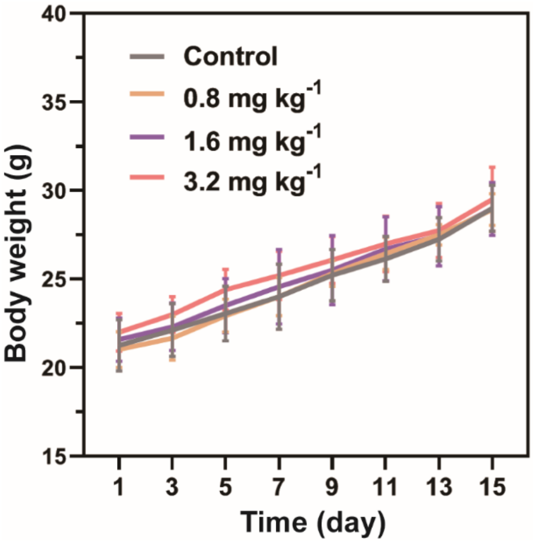

Figure S22. Body weights of mice in different groups, including Control, 0.8 mg kg^{-1}, 1.6 mg kg^{-1} and 3.2 mg kg^{-1}.

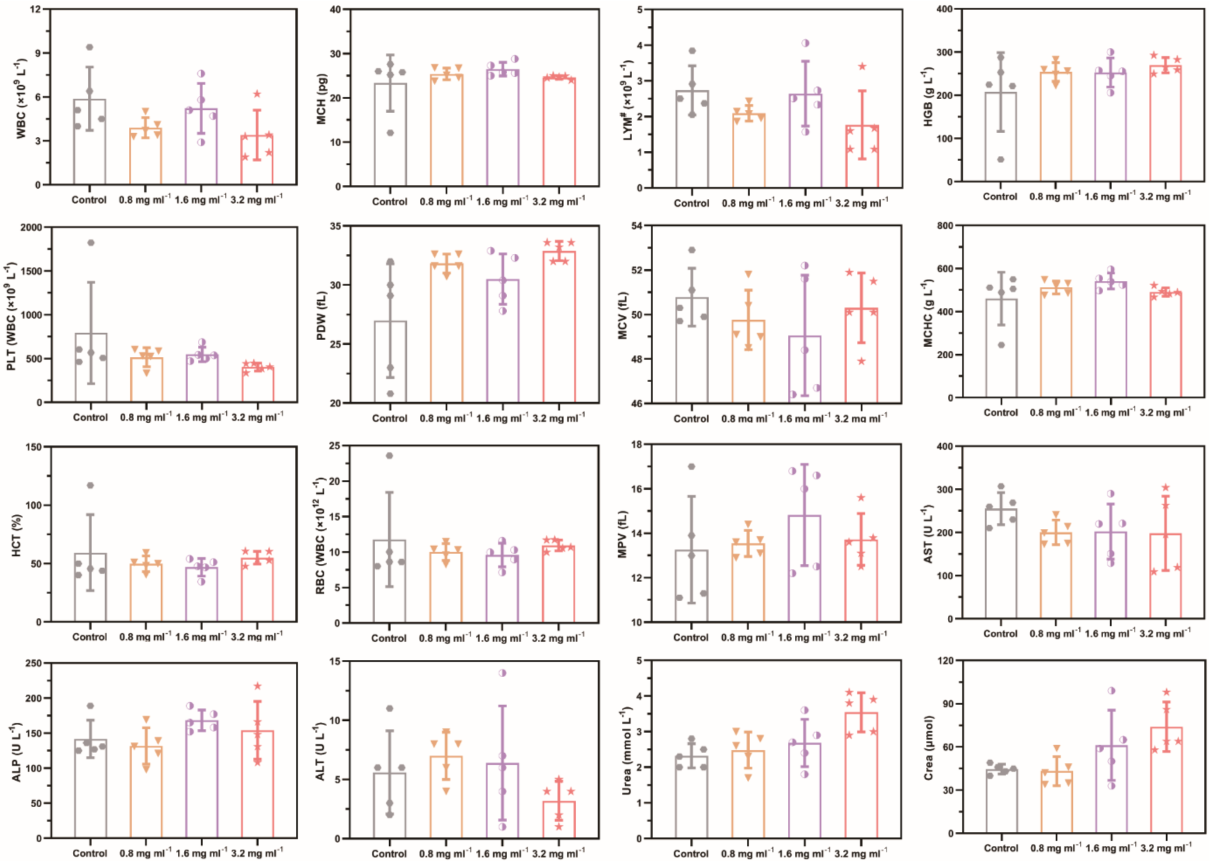

Figure S23. Blood routine and blood biochemical evaluation of mice on the 15th day in different groups, including Control, 0.8 mg kg^{-1}, 1.6 mg kg^{-1}, and 3.2 mg kg^{-1}.

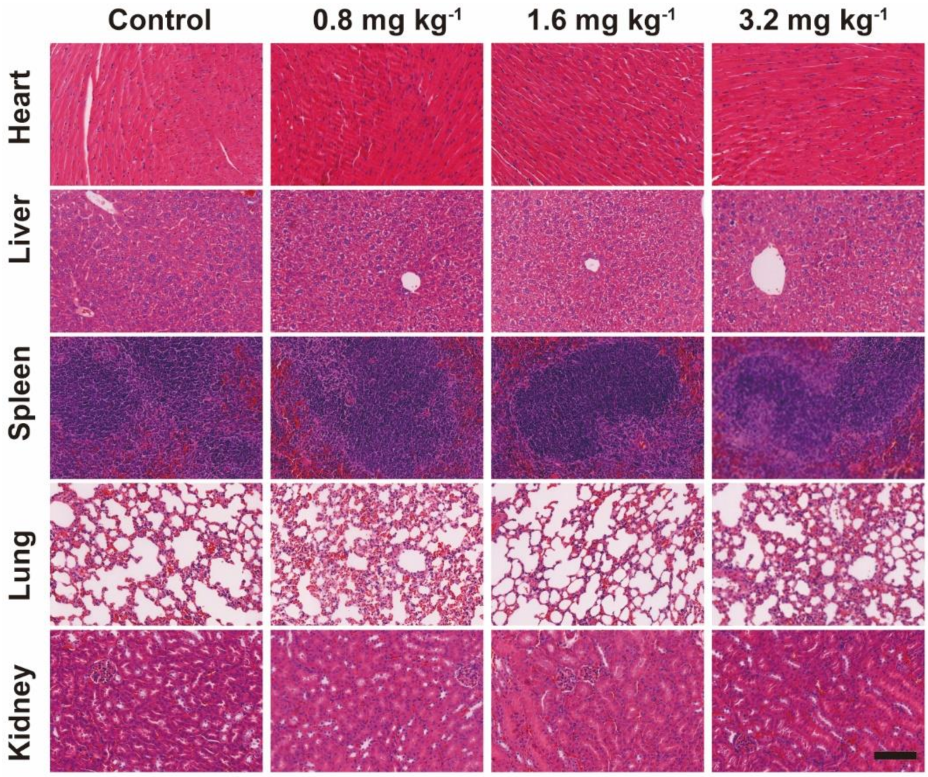

Figure S24. Hematoxylin & eosin (H&E) stained major organs (heart, liver, spleen, lung and kidney) in different groups, including Control, 0.8 mg kg^{-1}, 1.6 mg kg^{-1} and 3.2 mg kg^{-1}. Scale bar: 100 μm.

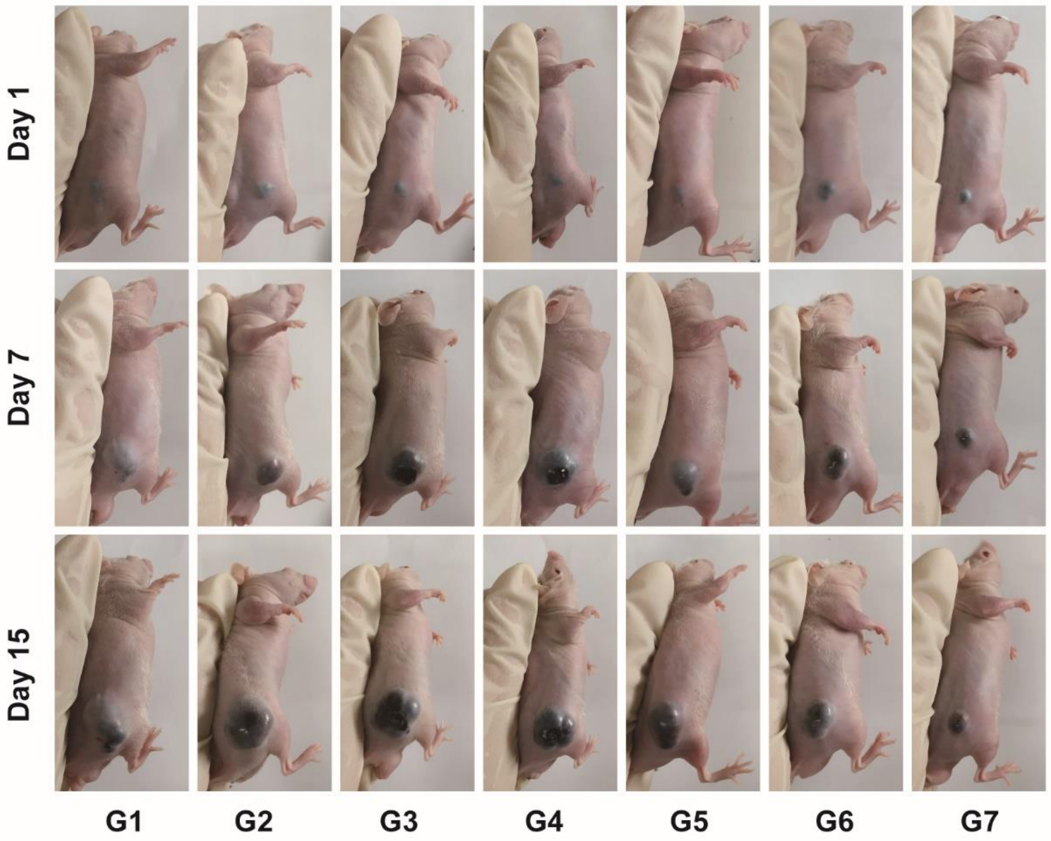

Figure S25. Digital pictures of B16F10 tumor-bearing mice after different treatments for 1, 7, and 15 days. The groups were: (G1) Control, (G2) Gel, (G3) Fe-C_3N_4-Gel, (G4) US, (G5) Gel + US, (G6) C_3N_4-Gel + US, and (G7) Fe-C_3N_4-Gel + US.

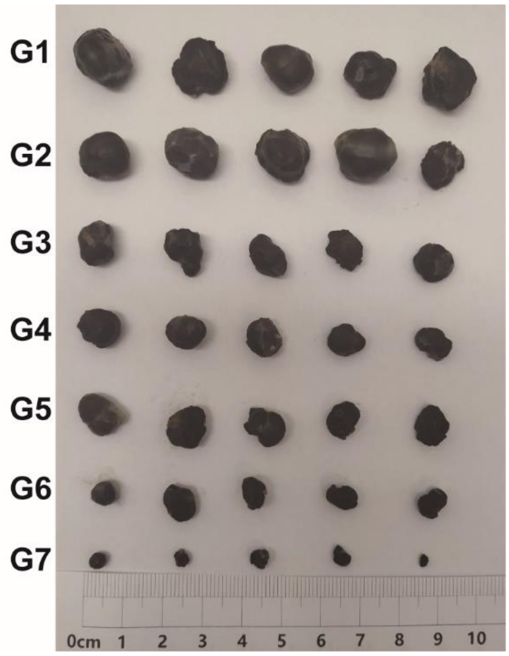

Figure S26. Photograph of B16F10 tumors dissected from tumor-bearing mice after different treatments on the 15th day. The groups were: (G1) Control, (G2) Gel, (G3) Fe-C_3N_4-Gel, (G4) US, (G5) Gel + US, (G6) C_3N_4-Gel + US, and (G7) Fe-C_3N_4-Gel + US.

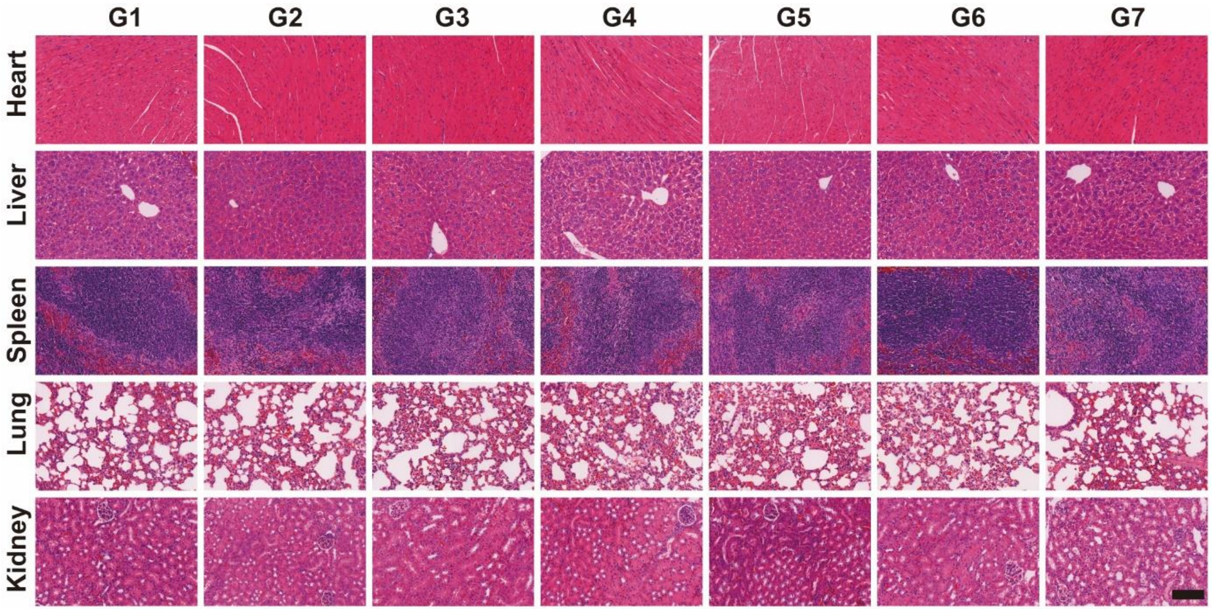

Figure S27. H&E-stained tissue sections of major organs (heart, liver, spleen, lung, and kidney) collected from mice injected with different treatments. The groups were: (G1) Control, (G2) Gel, (G3) Fe-C_3N_4-Gel, (G4) US, (G5) Gel + US, (G6) C_3N_4-Gel + US, and (G7) Fe-C_3N_4-Gel + US. Scale bar: 100 μm.

References

[1] S. An, G. Zhang, T. Wang, W. Zhang, K. Li, C. Song, J. T. Miller, S. Miao, J. Wang, X. Guo, *ACS Nano* **2018**, *12*, 9441.

[2] X. Zhang, X. Xie, H. Wang, J. Zhang, B. Pan, Y. Xie, *J Am Chem Soc* **2013**, *135*, 18.

[3] a) M. Ge, D. Xu, Z. Chen, C. Wei, Y. Zhang, C. Yang, Y. Chen, H. Lin, J. Shi, *Nano Lett* **2021**, *21*, 6764; b) P. Zhu, Y. Chen, J. Shi, *Adv Mater* **2020**, *32*, e2001976; c) M. Rahmati, P. B. Milan, A. Samadikuchaksaraei, V. Goodarzi, M. R. Saeb, S. Kargozar, D. L. Kaplan, M. Mozafari, *Macromol Mater Eng* **2017**, *302*, 1700227.

[4] G. Kresse, J. Furthmuller, *Phys Rev B* **1996**, *54*, 11169.

[5] J. P. Perdew, K. Burke, M. Ernzerhof, *Phys Rev Lett* **1997**, *77*, 3865.

[6] a) S. Grimme, J. Antony, S. Ehrlich, H. Krieg, *J Chem Phys* **2010**, *132*, 154104; b) S. Grimme, S. Ehrlich, L. Goerigk, *J Comput Chem* **2011**, *32*, 1456.

[7] a) S. Anders, P. T. Pyl, W. Huber, *Bioinformatics* **2015**, *31*, 166; b) A. Roberts, C. Trapnell, J. Donaghey, J.L. Rinn, L. Pachter, *Genome Biol* **2011**, *12*, R22.

[8] a) S. Anders, W. Huber, *European Molecular Biology Laboratory*, Heidelberg, Germany **2019**; b) M. I. Love, W. Huber, S. Anders, *Genome Biol* **2014**, *15*, 550; c) M. Kanehisa, M. Araki, S. Goto, M. Hattori, M. Hirakawa, M. Itoh, T. Katayama, S. Kawashima, S. Okuda, T. Tokimatsu, Y. Yamanishi, *Nucleic Acids Res* **2008**, *36*, D480.
